# Effectiveness of acupuncture as adjunctive therapy in type 2 diabetic: Study protocol for a randomized controlled trial

**DOI:** 10.1371/journal.pone.0284337

**Published:** 2023-09-20

**Authors:** Yean Chin Cheok, Zalilah Mohd Shariff, Yoke Mun Chan, Ooi Chuan Ng, Ping Yein Lee

**Affiliations:** 1 Department of Nutrition and Dietetics, Faculty of Medicine and Health Sciences, Universiti Putra Malaysia, Selangor, Malaysia; 2 Department of Medicine, Faculty of Medicine and Health Sciences, Universiti Putra Malaysia, Selangor, Malaysia; 3 Department of Family Medicine, Faculty of Medicine and Health Sciences, Universiti Putra Malaysia, Selangor, Malaysia; Centre Hospitalier Sud Francilien, FRANCE

## Abstract

**Introduction:**

The incidence of type 2 diabetes mellitus is increasing worldwide. The literature suggests that acupuncture is a possible complementary therapy for type 2 diabetes mellitus. This study aims to determine the effectiveness of acupuncture as an adjunctive therapy on homeostasis model assessment-insulin resistance (HOMA-IR), and health-related quality of life (HRQoL) in patients with type 2 diabetes mellitus.

**Materials and methods:**

This randomized, double-blind, placebo controlled, and parallel design trial will be carried out in a public university teaching hospitals in Malaysia. Eligible type 2 diabetes mellitus subjects will be randomly assigned to receive either acupuncture (n = 30) or a placebo (n = 30). The intervention is carried out using press needle or press placebo on abdomen area (10 sessions of treatment). Both groups will continue with their routine diabetes care. Primary outcome of HOMA-IR will be measured at the time of recruitment (-week 0), and after completion of 10 sessions (week 7) of the treatment. Additionally, secondary outcome of HRQoL will be measured at the time of recruitment (-week 0), after completion of 5 sessions (week 3/4), and 10 sessions (week 7) of the treatment. Any adverse event will be recorded at every visit.

**Discussion:**

The findings of this study will provide important clinical evidence for the effect of acupuncture as adjunctive therapy on HOMA-IR, adiposity and HRQoL of type 2 diabetes mellitus.

**Trial registration number:**

NCT04829045.

## Introduction

Globally, approximately 537 million people are living with diabetes mellitus [1] with most are type 2 diabetes mellitus [2, 3]. Type 2 diabetes mellitus involves a decline in b-cell function and increase in insulin resistance [4, 5]. Long term insulin resistance is associated with many complications [6, 7] which will adversely affect quality of life [8]. Currently, among the several mechanism-based drugs available for treatment of type 2 diabetes mellitus, only insulin sensitizers such as biguanides and thiazolidinediones have direct effects on insulin resistance [9, 10]. However, pharmacological agents have limitations related to patient’s poor adherence or resistance to intensify treatment and clinical inertia [5, 11]. These may be associated with the fear of side-effects of the medications [11]. Complementary and alternative medicine (CAM) may provide a beneficial adjunctive therapy for diabetes mellitus due to its perceived less adverse effect and culturally more acceptable in Asian population. Among the CAM, acupuncture is one of the most widely used combined therapies with conventional treatment for type 2 diabetes mellitus [12]. Additionally, the United States National Institutes of Health (NIH) has acknowledged that acupuncture can be used as adjunctive treatment for diabetes mellitus [13] with minimum adverse effect [14, 15] by a well-trained practitioner [16].

Acupuncture is a technique involving the insertion of very fine needles into specific points to affect the flow of body’s *qi* [17], or vital energy. According to the theory of traditional Chinese medicine (TCM), acupuncture can regulate *qi* and blood and have a direct impact on bioavailability of substances of the body [18]. In the past 20 years, several randomized controlled trials (RCTs) have been conducted on the effectiveness of acupuncture on diabetes mellitus and its complications [19–21]. It has been demonstrated that acupuncture may improve insulin sensitivity [18, 22] and c-peptide levels through effective treatment against metabolic disturbances such as hyperglycaemia [21, 23, 24], overweight [24, 25], inflammation [26], while improving lipid metabolism [27]. Equally, systematic reviews have also confirmed the benefits of acupuncture in patients with diabetes mellitus [28–30]. In addition, acupuncture could control co-morbidity conditions such as obesity [31, 32], pain [33, 34], anxiety, depression [35], stress [36], sleeping disorder [37, 38] and hence improves quality of life [39, 40]. However, multiple methodological flaws with substantial risk of bias have been identified in many of these RCTs [40, 41]. Therefore, this double-blind, placebo controlled clinical trial is designed to investigate the effectiveness of acupuncture as adjuvant therapy in patients with type 2 diabetes mellitus in accordance with the Consolidated Standards for Reporting of Trials Statement 2010 (CONSORT 2010) [42] and revised Standards for Reporting Interventions Controlled Trials of Acupuncture 2010 (STRICTA 2010) [43] guidelines.

## Materials and methods

### Objectives

This study aims to determine the effectiveness of acupuncture treatments on homeostasis model assessment-insulin resistance (HOMA-IR), and health-related quality of life (HRQoL) in patients with type 2 diabetes mellitus.

### Trial design

This is a randomized, double-blinded (patients and practitioners), placebo and two-arm parallel controlled clinical trial. This study will be conducted at a public university teaching hospital in Malaysia. The reporting of the protocol will adhere to the Standard Protocol Items: Recommendations for Interventional Trials (SPIRIT) [44] (S1 Table; S1 File) and STRICTA [43] guidelines (S2 Table).

### Ethics approval

The trial protocol has been approved by the Universiti Putra Malaysia Ethics Committee for Research Involving Human Subjects (JKEUPM-2018-294) (S2 File), and registered in ClinicalTrial.gov (NCT04829045). The study is performed according to Declaration of Helsinki and the guidelines for Good Clinical Practice. Permission to carry out the study will be obtained from the relevent hospital before conducting the research.

### Sample-size calculation

Based on the previous study by Firouzjaei et al. [18], it was assumed that the mean differences of HOMA-IR and standard deviation (SD) between the acupuncture group and control group were 1.23 and 1.25, respectively. For the mean comparison method of two-sample t-test model, a significance level of 5% z_(1-∂/2)_ = 1.96 and a power of 90% z_(1-ß)_ = 1.28 will be utilized to calculate the required sample size.


$$\begin{array}{ll} n & =\frac{2\times {\left(Z_{\alpha }+Z_{\beta }\right)}^{2}\times \sigma^{2}}{\delta^{2}}\\ & =\frac{2\times {\left(1.96+1.28\right)}^{2}\times {\left(1.25\right)}^{2}}{{\left(1.23\right)}^{2}}=21.68 \end{array}$$

For equal allocation of the two groups, the total sample size required when considering a dropout rate of 30% shall be 60 participants, with 30 participants in each group.

### Methods of recruitment

Subject recruitment is conducted at a public university teaching hospital in Malaysia by an endocrinologist and a TCM practitioner through patients’ routine medical check-up schedules. Patients will be provided study brochure and information sheet if they express interest to participate in the study. Patients are requested to fill up a screening form to assess the eligibility. For screening purpose, type 2 diabetes mellitus is defined as one abnormal fasting plasma glucose (FPG) ≥ 7.0 mmol/L (126 mg/dL), random venous plasma glucose ≥ 11.0 mmol/L (200 mg/dL), two hour plasma glucose measurement ≥ 11.0 mmol/L (200 mg/dL) or glycated haemoglobin (HbA1c) ≥ 6.5% based on subjects’ self-reported or through their diabetes mellitus book, medications packages, and clinic follows up cards. Potential subjects will be invited for a blood test screening session. Type 2 diabetes mellitus is confirmed with subject’s HbA1c ≥ 6.5% or morning FPG ≥ 7.0 mmol/L (126 mg/dL)(fasting at least 10 hours) based on American Diabetes Association recommendation [45]. Patients are required to sign a written informed consent form once they fulfil all the study criteria and agree to participate in the study.

### Eligibility criteria

The subject eligibility criteria for the study include Malaysian, age between 30–69 years old, have had type 2 diabetes mellitus (FPG ≥ 7.0 mmol/L [126 mg/dL] or HbA1c ≥ 6.5% for more than one year and with body mass index (BMI) ≤ 40.0 kg/m^2^; receive oral anti-diabetic agents on a stable dose over the previous 3 months. Patients with any of the following criteria will be excluded: 1) under insulin therapy; 2) with other acute or chronic health problems (eg. chronic kidney disease [stage 4 and 5]/heart/liver failure, cancer, cardiovascular disease, stroke, physical disability, mental illness, nephrotic syndrome, decompensated congestive heart failure and oedema on the abdomen); 3) needle phobia or allergy to adhesive plaster; 4) planning to move out from Malaysia within 4 months’ and 5) being pregnant, planning for pregnancy or lactating women.

### Randomization and allocation strategy

Blocked randomization with block size of 2 will be performed in a 1:1 allocation ratio to minimise between-group differences on HOMA-IR level and BMI. The press needles and placebos are identical in appearance. They are repacked in similar plastic containers and consecutively numbered for each subject according to the randomization schedule. Each subject is assigned an order number. The containers will be distributed to the principle investigator at the site and practitioner will receive the corresponding containers. Randomization sequence and allocation will be concealed to all subjects, acupuncturist, laboratory personnel and researchers. Sequentially numbered, sealed opaque envelopes will be used to conceal treatment procedures. The randomisation sequence will remain concealed until the end of the study.

### Compliance

Adherence to the protocol is assessed at every follow-up session, and evaluation of intervention adherence will be assessed at post intervention.

### Intervention

Subjects will receive 10 sessions of acupuncture treatment using press needles or press placebos. During the treatments, all subjects are required to take supine position. The acupuncture points will be disinfected with a disposable 70% isopropyl alcohol cotton. After all the devices are fixed, acupoints will be pressed for at least three times (at 0, 15 and after 30 minutes) for about 30 seconds, with the degree that can be tolerated by the subject. The press needle or placebo is inserted according to the acupoints selection sequence, the product directions and following same procedures [46]. The validity and credibility of the device have been well demonstrated in other studies [47, 48].

In this study, an irradiating needling sensation (*de qi*) does not have to be achieved. *De qi* is a tingling, numbness, and heaviness feelings that occur after an acupuncture needle had been inserted in the body [49]. During the 45 minutes device retention period, subject’s abdomen will be covered to prevent exposure. If the subjects are sick or not feeling well on that day, they will not be given any treatment and are requested to come on other day. Likewise, if subject reports discomfort or encounter any adverse effect during intervention, therapy is discontinued and the treatment for that particular session will not be included.

**Fig 1:**
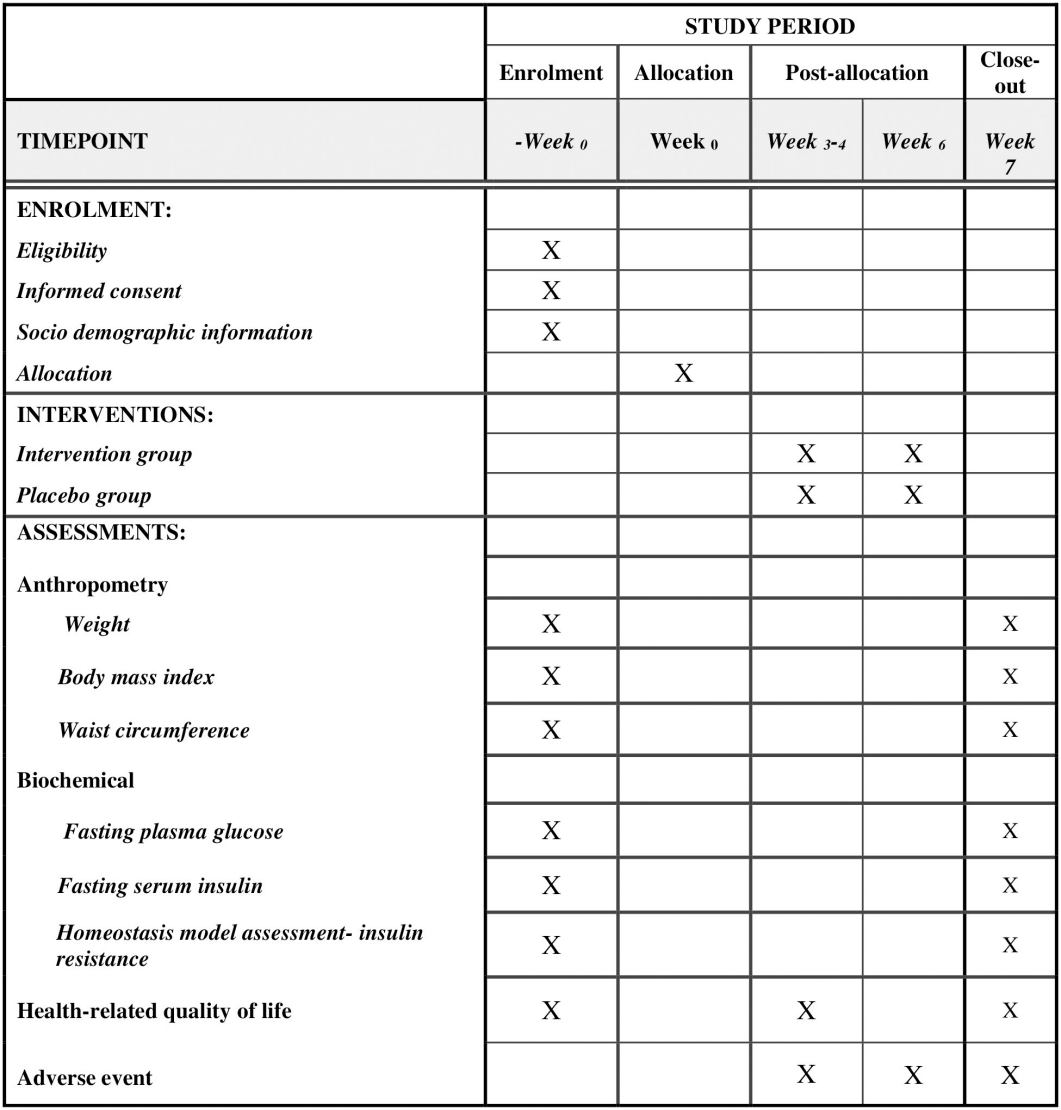
SPIRIT schedule of enrolment, interventions, and assessments.

The study period is 7 weeks with intervention period for 6 weeks and follow-up for any adverse event for 1 week (Fig 1). The participants will be treated twice weekly in the first four weeks then once a week for the next two weeks. In certain circumstances, once-a-week treatment or a three-time per week treatment is permitted, but 10 treatments must be completed within 6 weeks. For those who are under drug(s) or other treatments during intervention, they are required to continue with their existing treatment regimens. All medication and doses received by the subjects are recorded and monitored during the course of the study to ensure the drugs are maintained throughout the study. All pharmacology agents that are allowed in this study are oral anti-diabetic drugs, hypertensive and hyperlipidaemia medications. Medication that are not allowed in this study are all the insulin; all the Glucagon-like peptide-1 receptor agonists such as Liraglutide, Exenatide, Dulaglutide, Semaglutide, and thiazolidinediones (for example: Pioglitazone and Rosiglitazone). There will be no change in other treatment as long as patients are clinically stable. Subjects are also advised to notify the practitioners immediately if there are any changes in their current treatments/circumstances such as personal details, medication or pregnancy (for woman) during the study. Subject’s is considered drop-outs if they have missed their appointments for more than three times, they do not attend the baseline or assessment session (cause missing data), they become pregnant (for women), inability to comply with protocol requirements, withdrawal of his/her own decision or advised by the researcher (for example participant increase 20% doses of diabetes mellitus medication or using acupuncture at other clinic during intervention). Any changes in the treatment regimen will be documented. Safety or adverse event in each session will also be recorded in a case record form.

#### Treatment group

The treatment group will receive real acupuncture treatments using new, disposable, sterilized, stainless-steel press needles (PYONEX ø0.20×1.5mm made by Seirin Corporation). A standardized acupoints formula with 6–10 needles on abdomen area such as Zhongwan (Ren-12), Xiawan (Ren-10), Qihai (Ren-6), Guanyuan (Ren-4), Tian Shu (St-25), Daheng (Sp-15), Shangqu, Jinhe (M-HN)/Qixue (Kd-13), Shuidao (St-28) and Liang Men (St-21) are selected based on the theory of abdominal acupuncture of CAM, the literature reviews [18, 22, 50, 51], and recommendation by international experts. The compulsory acupoints for all participants are Ren 12, Ren 10, Ren 6 (men) or Ren 4 (women), and ST25; others acupoints will be incorporated based on the four diagnostic criteria of the TCM which includes observation, smelling, listening, inquiring, and palpations [52]. The number and name of acupuncture points used in each session will be documented.

#### Control group

**Fig 2:**
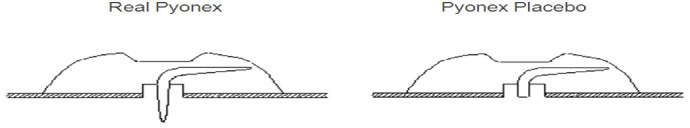
Press needle and press placebo.

Subjects will receive the same treatment as in the intervention group. However, subject is given press placebos (made by Seirin Corporation) which is identical to the press needle in all aspects except the needle element is lacking [48, 53] (Fig 2).

### Practitioner’ background

Interventions are administered by a qualified TCM practitioner and acupuncturist. She has a master’s degree in a relevant field and with significant clinical experience. She has been a member of British Acupuncture Council, United Kingdom since 2012. She has attended abdominal acupuncture seminar in year 2008.

### Measurements

Socio-demographic backgrounds of patients which include patients’ age, gender, ethnicity, marital status, educational level, personal monthly income, duration of diabetes mellitus, and family history of diabetes mellitus will be obtained through a questionnaire at recruitment. The primary outcome and secondary outcomes of body mass index (BMI), and waist circumference will be measured at baseline (-week 0), and after the treatment (week 7). The secondary outcome of HRQoL will be measured at baseline, during and after the treatment is completed. Data will be collected at the time of recruitment (-week 0), after completion of 5 sessions (week 3/4) and 10 sessions (week 7) of the treatment.

#### Primary outcome

The primary outcome of this trial is the change in HOMA-IR between and/or within group(s) after treatment. Fasting insulin and glucose concentrations will be used to derive the HOMA-IR [fasting serum insulin (μU/ml)×fasting plasma glucose (mmol l-1)/22.5] [54]. Fasting insulin levels and fasting glucose will be analyzed using heparin tubes and sodium fluoride tubes respectively by an enzyme-linked immunosorbent assay, also called ELISA or EIA. Peripheral blood will be drawn from the antecubital veins of the subjects at baseline and at week 7 after a 10 hour overnight fasting. A total of 10 ml/L of venous blood will be obtained using venipuncture method. These blood samples are then centrifuged for 10 minutes at the speed of 3500rpm to separate the blood serum. Subsequently, serum will be put in an Eppendorf tubes for preservation. The serum is preserved in a low temperature freezer which shall maintain at a temperature of -70 Celsius.

#### Secondary outcomes

The secondary outcomes are changes in HRQoL. The HRQoL of subject will be assessed using a validated World Health Organization Quality of Life Assessment: Brief Version (WHOQoL-BREF [English/Malay]) questionnaire [55, 56]. Anthropometry measurements will be measured according to the standard procedure. Subjects will be measured for weight in kilogram to the nearest 0.1 kilogram, using a digital scale TANITA model HD-382 weighing machine in a standing position. Height will be measured using SECA body meter model 208 to the nearest 0.05 centimetre. Body mass index is calculated weight in kilogram divided by height in meters squared (BMI = Weight(kg)/Height^2^(m^2^). Waist circumference will be measured midway between the lowest rib and the iliac crest using SECA measuring tape to the nearest 0.05 centimetre. Central obesity for Asian population is defined as having a waist circumference ≥ 90cm for men and ≥ 80cm for women based on WHO/IASO/IOTF criteria [57].

### Statistical analysis

Data will be analyzed using SPSS Statistics 26.0 (IBM Corp., New York, USA). Comparisons of baseline continuous variables between acupuncture and control group will be examined using independent *t* test, while for categorical variables the Chi-squared test will be used. The primary outcome is the change in HOMA-IR from baseline to 7 weeks. The secondary outcome measure is the change of HRQoL from baseline to week 3/4 and week 7. This will be done using a linear mixed model. The model included treatment group (acupuncture or control), time (baseline, week 3/4, and week 7), age, gender, and their interaction as fixed effects. Random intercepts for each subject are also being included in the model to account for individual variability within the data. Intention-to-treat analysis (ITT) will be performed, and all patients enrolled will be included in the analysis, irrespective of compliance. The significant level is set at P<0.05.

### Safety assessments

Safety or adverse event suspected related to the treatment, through the symptoms reported by the patients, and observations by researcher at every visit are collected. Adverse event is defined as any unfavourable and unintended sign, symptom, or disease temporally associated with the use of a medical treatment or procedure that may or may not be considered related to the medical treatment or procedure [49]. Possible adverse events related to acupuncture are bruising, nausea, temporary pain, dizziness or faintness, or discomfort [58]. Details about the event time, severity, and duration, causal relationship with the treatment, other treatments or medications that are suspected to cause the adverse event will be recorded.

### Data management

Data will be entered into computer by a study investigator (Yean Chin Cheok). All study-related physical files will be stored in a secure place. The data will be kept at Setia Chinese Medical Centre for five years after the data collection.

### Quality assurance

To maintain the overall quality and legitimacy of the clinical trial, code breaks should occur only upon the occurrence of adverse event occurred. The site investigators (Yean Chin Cheok, Ooi Chuan Ng) must record all code breaks with reason on the corresponding CRF. To ensure the practitioner is masked to the group allocation, she is requested not to check for the presence of a sharp tip below the plaster during the intervention. She will also be briefed about risk management of the trial during the training. The Ethics Committee for Research Involving Human Subjects of Universiti Putra Malaysia (JKEUPM), Universiti Putra Malaysia will conduct random trial audit, if necessary.

## Discussion

Diabetes mellitus is increasing rapidly globally, particularly in low-and middle-income countries [59]. According to the International Diabetes Federation (IDF), Malaysia had an estimated 4.4 million adults aged between 20–79 living with diabetes in 2021, making it one of the countries with the highest rates of diabetes mellitus in the world [60]. This represents a prevalence rate of approximately 19.0% among adults in Malaysia [60], with the Indians having the highest prevalence of diabetes mellitus at 18.5%, followed by the Malays (11.0%), Chinese (8.5%), and Bumiputra Sarawak (7.9%) [61]. It is estimated that up to 49.0% of people with diabetes mellitus in Malaysia were undiagnosed, which means they were not aware of their condition and were not receiving treatment [60]. In addition, it has been estimated that 15.5% of the Malaysian population had impaired glucose tolerance [60]. Impaired glucose tolerance is a condition where blood glucose levels are higher than normal but not high enough to be classified as diabetes mellitus. Diabetes mellitus increased the risk of cardiovascular diseases, and substantially accounted for 35% in total Malaysian mortality [62]. Type 2 diabetes mellitus was historically considered to be a disease that primarily affected middle-aged and elderly people. However, in recent years, there has been a rising prevalence of the disease among children and young people in Malaysia. The prevalence of type 2 diabetes mellitus among children and young people under the age of 24 in Malaysia was estimated to be around 2.81% [63], which is a significant increase from previous estimates. By 2025, diabetes mellitus is expected to affect 7 million Malaysian adults aged 18 and older [64]. It is reported that the major risk factors for diabetes mellitus in Malaysia include physical inactivity (51.6%), overweight (37.3%) and obesity (12.9%) [65].

Obese people have high levels of non-esterified fatty, acids, cytokines, glycerol, pro-inflammatory markers, hormones and other substances that are actively involved in development of insulin resistance [66]. In 2019, it was revealed that one in two adults in Malaysia was overweight, obese or with abdominal obesity [67]. By genders and ethnicity, more females than males were overweight or obese (54.7%), and had abdominal obesity (64.8%) while Indian has the highest prevalence of overweight or obese (63.9%), and abdominal obesity (68.3%). Apart from that, 55–59 years old age group had the highest prevalence of overweight or obesity (60.9%) whereas 60–64 years old age group had the highest prevalence of abdominal obesity (71.5%) [67]. Therefore, the increasing trend of overweight to a concomitant rise of type 2 diabetes mellitus is expected in Malaysia. Several studies have reported that acupuncture may improve insulin sensitivity [22, 27] and is effective against metabolic disturbances including obesity [32].

Acupuncture has been utilized extensively for the treatment of many different medical conditions [68]. Abdominal acupuncture, a traditionally based system of acupuncture is an innovative treatment in the field of CAM in which acupuncturists choose specific abdomen acupoints to treat various diseases [69]. Abdominal acupuncture is believed to be able to activate Prenatal or Congenital Channel System that radiates from the umbilicus [70]. An important characteristic of the system is that it is very close to the surface of the abdominal wall [70]. Hence, needling is performed on a more superficial level [70]. The mechanism by which acupuncture helps to reduce obesity and insulin resistance remains unclear. As such, it is pertinent to determine the treatment effect of acupuncture which may offer more insights on the prevention and management of patients with type 2 diabetes mellitus.

Treatment with shallow insertion using press needles have been described and recommended [48, 53]. It is generally more practical, safe and scientifically approved as they are away from internal organ [53]. Press needle with shallow insertion can reduce the side effects of pain, traumatic, bleeding, haematoma, or pneumothorax during intervention [53]. Safety is one of the most important issues in the use of acupuncture in clinical practice, especially for diabetes mellitus. Nevertheless, according to various studies in China, Korea, Japan, Germany and the United Kingdom, serious adverse event associated with acupuncture is rare [14, 15, 71] where the incidence rate is about 0.004%. Therefore, acupuncture appears to be a safe modality to complement the existing therapy for type 2 diabetes mellitus.

Diabetes mellitus is a significant healthcare burden worldwide, and the annual global healthcare spending on diabetes mellitus was estimated to be around USD 966 billion in 2021 [72]. Similarly, the economic burden of diabetes mellitus in Malaysia is high. The estimated cost of diabetes mellitus in Malaysia is around 600 million USD per year [73]. The high cost of diabetes mellitus places a significant challenge on healthcare systems, as resources are required to manage and treat the disease effectively. Therefore, this is the first double-blind, placebo controlled, acupuncture clinical trial in Malaysia to investigate the effectiveness of acupuncture as adjuvant therapy in patients with type 2 diabetes mellitus. The findings of this study can be used to develop evidence-based recommendations of acupuncture as adjunct therapy on HOMA-IR and/or HRQoL of type 2 diabetes mellitus patients, and will contribute to our understanding of the mechanisms of acupuncture in relation to type 2 diabetes mellitus.

## Supporting information

S1 Table SPIRIT 2013 checklist: Recommended items to address in a clinical trial protocol and related documents*. (DOC)

S2 Table STRICTA 2010 checklist. (TIF)

S1 File Trial registration data. (TIF)

S2 File Study protocol. (DOCX)

S1 Checklist SPIRIT 2013 checklist. (DOCX)
