# Supplementary material for: Effectiveness of acupuncture as adjunctive therapy in type 2 diabetic: Study protocol for a randomized controlled trial
Source: PLoS One. 2023 Sep 20;18(9):e0284337. doi: 10.1371/journal.pone.0284337 (PMC10511073; doi:10.1371/journal.pone.0284337)
Supplement: S1 Table — (DOC) [file pone.0284337.s001.doc]

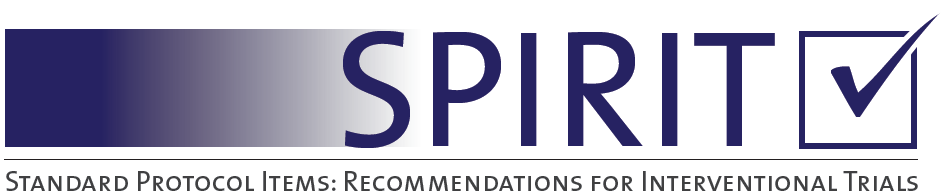


SPIRIT 2013 Checklist: Recommended items to address in a clinical trial protocol and related documents*

| Section/item | ItemNo | Description |
| --- | --- | --- |
| **Administrative information** | | |
| Title | 1 | A randomized, investigator and patient-blinded, 7 weeks, parallel-group study protocol to compare the efficacy of acupuncture versus placebo control group as adjunctive therapy in type 2 diabetic. |
| Trial registration | 2a | UTN: U1111-1219-3545  ClinicalTrials.gov identifier: NCT04829045  Institute Approval Letter: JKEUPM-2018-294 |
|  | 2b | See S1 File |
| Protocol version | 3 | Issue date: 21 March 2019  Authors: Yean Chin Cheok, Mohd Shariff Zalilah, Yoke Mun Chan, Ping Yein Lee and Ooi Chuan Ng |
| Funding | 4 | Self-funding |
| Roles and responsibilities | 5a | Study: Yean Chin Cheok conceived of the study. Mohd Shariff Zalilah, Yoke Mun Chan and Ping Yein Lee initiated the study design. Yean Chin Cheok, Mohd Shariff Zalilah, Yoke Mun Chan and Ooi Chuan Ng contributed to study implementation. Manuscript: Yean Chin Cheok prepared original draft; Mohd Shariff Zalilah reviewed and finalized the manuscript. Yoke Mun Chan, Ping Yein Lee, and Ooi Chuan Ng read and approved the final version of manuscript. |
|  | 5b | The author(s) received no specific funding for this work. |
|  | 5c | Not relevant |
|  | 5d | Yean Chin Cheok, Mohd Shariff Zalilah, Yoke Mun Chan and Ping Yein Lee are responsible for the preparation of the study protocol, case record form (CRF) and relevant documents with Ooi Chuan Ng provided inputs for revision. All investigators will oversee the trial implementation. Yean Chin Cheok and Ooi Chuan Ng will assist in recruiting subjects, performing, and overseeing treatment at site. All investigators will contribute to any report/publication outcomes of this trial. |
| Introduction |  |  |
| Background and rationale | 6a | Page 1-2 |
|  | 6b | Page 8 |
| Objectives | 7 | Page 2 |
| Trial design | 8 | Page 2-3 |
| Methods: Participants, interventions, and outcomes | | |
| Study setting | 9 | Page 2 |
| Eligibility criteria | 10 | Page 4-5 |
| Interventions | 11a | Page 6-8 |
|  | 11b | Page 6-7 |
|  | 11c | Page 5 |
|  | 11d | Page 7 |
| Outcomes | 12 | Page 9-10 |
| Participant timeline | 13 | Page 6-7 and Figure1 |
| Sample size | 14 | Page 3-4 |
| Recruitment | 15 | Page |
| **Methods: Assignment of interventions (for controlled trials)** | | |
| Allocation: |  |  |
| Sequence generation | 16a | Page 5 |
| Allocation concealment mechanism | 16b | Page 5 |
| Implementation | 16c | Page 4 |
| Blinding (masking) | 17a | Page 5 |
|  | 17b | Page 7 and 11 |
| **Methods: Data collection, management, and analysis** | | |
| Data collection methods | 18a | Page 8-10 |
|  | 18b | Page 6 |
| Data management | 19 | Page 11 |
| Statistical methods | 20a | Page 10 |
|  | 20b | Not applicable |
|  | 20c | Page 10 |
| **Methods: Monitoring** | | |
| Data monitoring | 21a | Data monitoring is not needed as this is a postgraduate project (Yean Chin Cheok). Upon completion of data entry / analyses / interpretation, the data will be reported to meet thesis requirement. |
|  | 21b | The trial will not be stopped unless there is any serious adverse event that cause by the trial, if the risks are found to outweigh the potential benefits or when there is conclusive proof of positive and beneficial results. In this case, the investigators will discuss the results of interim-analysis with the Ethics Committee members for Research Involving Human Subjects of Universiti Putra Malaysia. |
| Harms | 22 | Page 7 and 11 |
| Auditing | 23 | The Ethics Committee for Research Involving Human Subjects of Universiti Putra Malaysia will conduct random trial audit, if necessary. |
| Ethics and dissemination | | |
| Research ethics approval | 24 | This protocol and the informed consent form have been approved by the Ethics Committee for Research Involving Human Subject of Universiti Putra Malaysia (JKEUPM-2018-294). |
| Protocol amendments | 25 | Not relevant |
| Consent or assent | 26a | Page 4 |
|  | 26b | Not relevant |
| Confidentiality | 27 | All study-related information and subject information that contain names or other personal identifiers will be stored at the study site with limited access. In order to maintain subject confidentiality, subject’s CRFs will be identified by a coded identification number only. |
| Declaration of interests | 28 | The authors have declared that no competing of interests exists. |
| Access to data | 29 | All investigators will have full access to the complete and final dataset. |
| Ancillary and post-trial care | 30 | Subjects enrolled in the study are covered by insurance for any non-negligent harm arises. They will be follow-up for at least 3 months after the intervention. |
| Dissemination policy | 31a | Data will be disseminated through publication in PhD thesis / journals and presentations at national / international seminars / conferences. |
|  | 31b | No |
|  | 31c | No |
| Appendices |  |  |
| Informed consent materials | 32 | Sample informed consent form |
| Biological specimens | 33 | Not relevant |

*It is strongly recommended that this checklist be read in conjunction with the SPIRIT 2013 Explanation & Elaboration for important clarification on the items. Amendments to the protocol should be tracked and dated. The SPIRIT checklist is copyrighted by the SPIRIT Group under the Creative Commons “[Attribution-NonCommercial-NoDerivs 3.0 Unported](http://www.creativecommons.org/licenses/by-nc-nd/3.0/)” license.
