# Supplementary figures and images for: Effectiveness of acupuncture as adjunctive therapy in type 2 diabetic: Study protocol for a randomized controlled trial
Source: PLoS One. 2023 Sep 20;18(9):e0284337. doi: 10.1371/journal.pone.0284337 (PMC10511073; doi:10.1371/journal.pone.0284337)

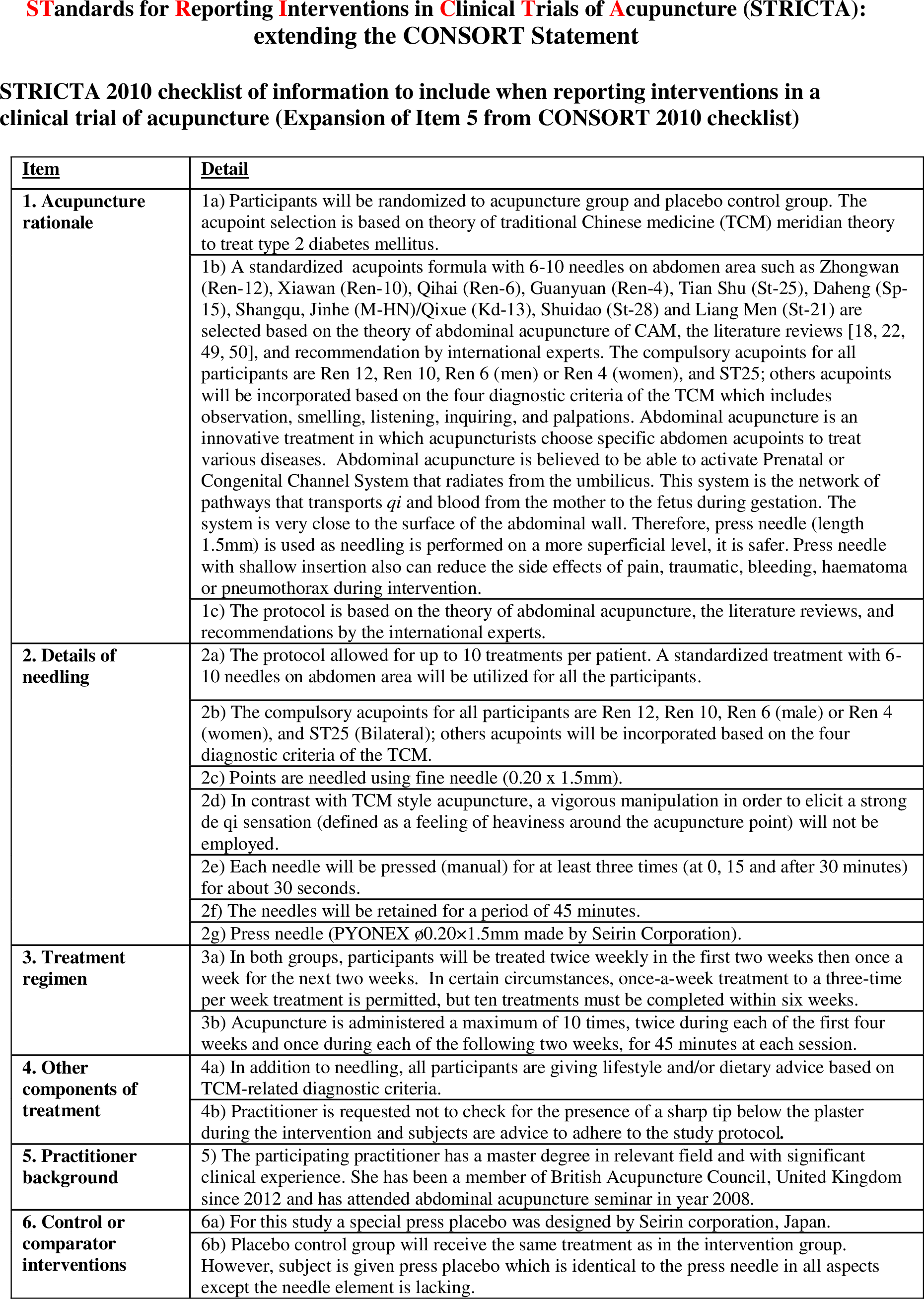

Supplement: S2 Table — (TIF) [file pone.0284337.s002.tif]

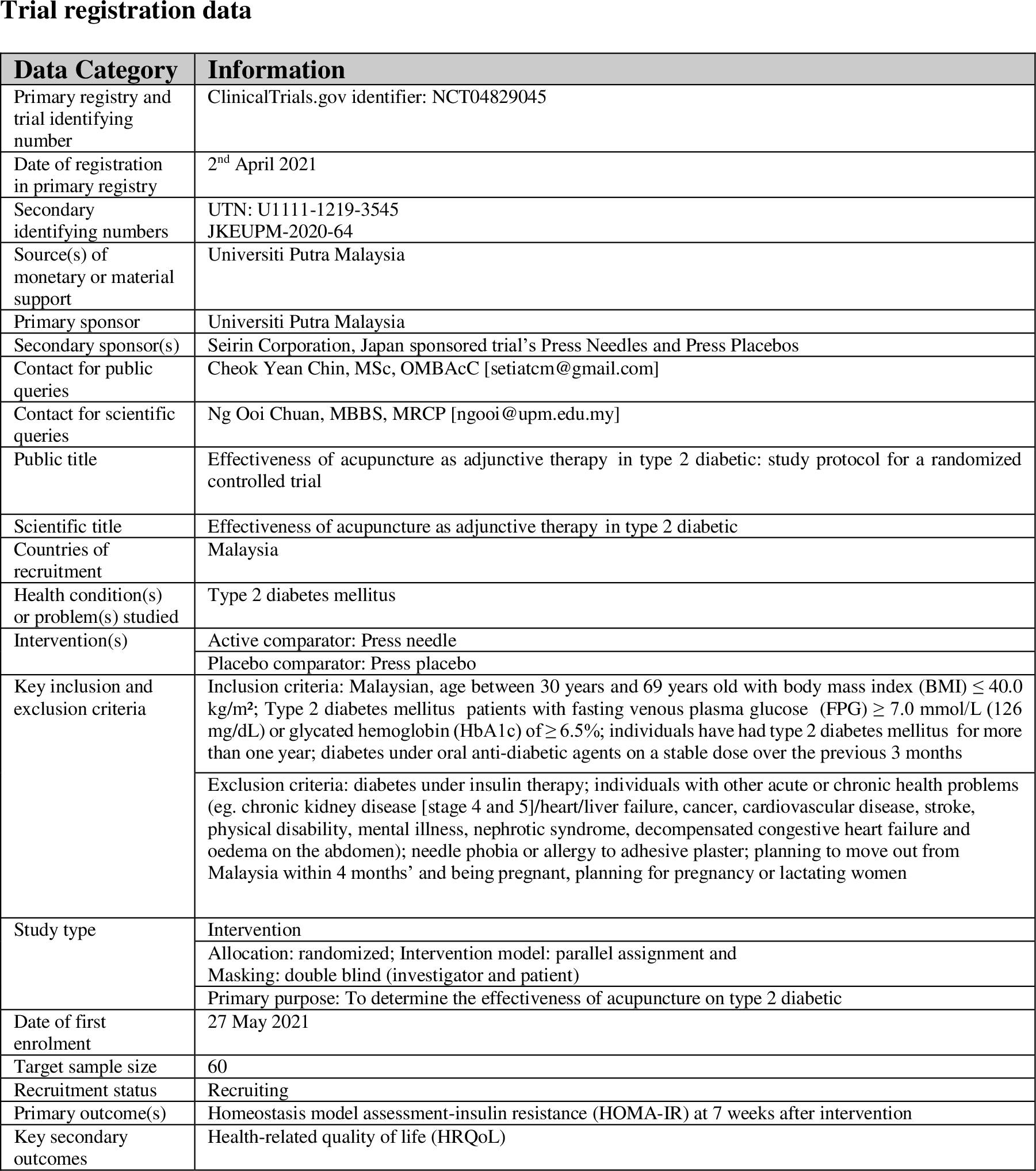

Supplement: S1 File — (TIF) [file pone.0284337.s003.tif]
