# Supplementary material for: Effectiveness of acupuncture as adjunctive therapy in type 2 diabetic: Study protocol for a randomized controlled trial
Source: PLoS One. 2023 Sep 20;18(9):e0284337. doi: 10.1371/journal.pone.0284337 (PMC10511073; doi:10.1371/journal.pone.0284337)
Supplement: S2 File — (DOCX) [file pone.0284337.s004.docx]

|  |
| --- |
| **THE EFFECT OF ACUPUNCTURE AS ADJUNCTIVE THERAPY ON HOMEOSTASIS MODEL ASSESSMENT-INSULIN RESISTANCE AND HEALTH-RELATED QUALITY OF LIFE IN PATIENTS WITH TYPE 2 DIABETES MELLITUS** |
| **UNIVERSITI PUTRA MALAYSIA (UPM)**  **FACULTY OF MEDICINE AND HEALTH SCIENCE** |
|  |
| **RESEARCH PROPOSAL (PhD Level)** |
|  |

|  |
| --- |

**SUPERVISORY COMMITTEE**

**Professor Dr. Zalilah Mohd. Shariff (Chairman)**

**Department of Nutrition and Dietetics**

**Faculty of Medicine and Health Sciences**

**Universiti Putra Malaysia**

**Professor Dr. Chan Yoke Mun (Member)**

**Department of Nutrition and Dietetics**

**Faculty of Medicine and Health Sciences**

**Universiti Putra Malaysia**

**Professor Dr. Lee Ping Yein (Member)**

**Department of Family Medicine**

**Faculty of Medicine and Health Sciences**

**Universiti Putra Malaysia**

**Dr. Ng Ooi Chuan, MBBS, MRCP (Member)**

**Department of Medicine**

**Faculty of Medicine and Health Sciences**

**Universiti Putra Malaysia**

**PREPARED BY:**

**Cheok Yean Chin**

**(GS44612)**

1. Introduction

## 1.1 Background

The global prevalence of diabetes has nearly doubled, from 4.7% in 1980 to 8.5% in 2014. Worldwide, approximately 463 million adults are living with diabetes mellitus (International Diabetes Federation [IDF], 2019) and most of them are type 2 diabetes mellitus (T2DM)(World Health Organization [WHO], 2019). Diabetes mellitus is increasing more rapidly in low-and middle-income countries than in high-income countries. South-East Asia and Western Pacific Regions have the largest number of people with diabetes mellitus, accounting for approximately half of the diabetes mellitus cases worldwide. An estimated 1.6 million deaths in 2015 were directly caused by diabetes mellitus (WHO, 2016) and it was estimated that the annual global health care spending on diabetes was US$ 850 billion in 2017 (IDF, 2017). It is a significant challenge for healthcare systems with such a high cost (IDF, 2015).

In Malaysia, diabetes mellitus is also a public health concern. The prevalence of diabetes mellitus among adults of 18 years and above has increased from 11.6% in 2006 to 17.5% in 2015 (Letchuman et al., 2010; Institute for Public Health [IPH], 2015) and is projected to be 21.6% by the year 2020 (Feisul and Azmi, 2013). There has also been an increasing prevalence of T2DM among adults aged ≥ 30 years in Malaysia, affecting 2.8 million individuals (Zanariah et al., 2015). The Indians had the highest prevalence of diabetes mellitus at 22.1%, followed by the Malays (14.6%) and Chinese (12.0%)(IPH, 2015). Diabetes mellitus has also been listed as one of the top 10 causes of premature mortality in Malaysia (Ummi et al., 2013).

T2DM is characterized by chronic hyperglycemia or high level of glucose in blood which stem from increased insulin resistance and insufficient levels of insulin (Olokoba, Obateru & Olokoba, 2012). People with long term hyperglycemic and insulin resistance will have increased risk of macrovasular and microvascular complications (IDF, 2015) and higher mortality (Holman, 2012). Complications of diabetes mellitus will adversely affect quality of life (Gupta, 2008) and reduce life span (Department of Health, 2012; Olivia et al., 2012; Podbielska et al., 2013; Cisneros et al., 2015).

Epidemiological experimental and clinical trials indicated that physical inactivity and some dietary practices are strongly associated with T2DM (Goedecke and Ojuka, 2014). It is estimated that more than 90% of diabetes mellitus cases could be prevented by engaging in moderate to vigorous physical activity for at least a half an hour per day, maintaining good nutrition, weight control, and smoking cessation (Morewitz, 2006). It is also demonstrated that physical activity may contribute to 30–50% reduction in the development of T2DM (Bassuk and Manson, 2005). Despite the importance of physical activity in the prevention of diabetes mellitus, physical inactivity remains the key reason for causing approximately 27% of diabetes mellitus cases in the world (WHO, 2013).

When lifestyle approach fails to maintain adequate glycemic control, pharmacological agents in the forms of oral, inhaled and injectable products are used to manage T2D (Charles, 1998; Ibrahim, 2010; Currie, 2012). However, these medications have limitations related to patient’s poor adherence (Currie et al., 2012; Gelaw et al., 2014; Reach, 2015; Kassahun et al., 2016), patient resistance to intensify treatment and doctors’ clinical inertia (Grant et al., 2011; Currie et al., 2012; Reach, 2015; Khunti et al., 2015). These limitations may be associated with the fear of side-effects of diabetes mellitus medication (Ruzaidi et al., 2008; Zafar et al., 2010; Khunti, 2015; Kassahun et al., 2016). Complementary and alternative medicine (CAM) may provide a beneficial adjunctive therapy for diabetes mellitus due to its perceived less adverse effect and culturally more acceptable in Asian population. Among the CAM, acupuncture is one of the most widely used combined therapies with conventional treatment for T2DM (Lin et al., 2013; Lin et al., 2014; Tjipto, 2014; Firouzjaei et al., 2016; Dong et al, 2017).

Acupuncture is a technique involving the insertion of fine needles into specific points on the body surface (British Acupuncture Council, 2019). It has been used for more than 2,500 years since its inception in China (VanderPloeg & Yi, 2009). It is an integral part of traditional Chinese medicine (TCM) or traditional medicine (TM). TM has a long history of use in health maintenance and in disease prevention and treatment, particularly for chronic disease (WHO, 2013). According to the theory of TCM, acupuncture can regulate *qi* and *blood* and have a direct impact on bioavailability of substances of the body (Firouzjaei et al., 2016). Acupuncture has become more established in conventional treatment as an alternative or complement medicine (Elisabet, 2008; Takahashi, 2009).

Acupuncture has been shown to be effective in treating diabetes mellitus in clinical randomized controlled trials (RCTs)(Yang and Liu, 2015; Firouzjaei et al., 2016). According to Tjipto, Saputra & Sutrisno (2014), acupuncture as adjunctive therapy on diabetes mellitus showed significant decreased in fasting plasma glucose (FPG)(P<0.01) and oral glucose tolerances test (OGTT)(P<0.001). Similarly, it had been demonstrated that acupuncture combined with metformin was more effective than metformin alone on homeostasis model assessment-insulin resistance (HOMA-IR)(Firouzjaei et al. 2016)(weight mean difference (WMD) -1.34, 95%CI -1.56, -1.12, P<0.001), which acting as an insulin sensitizer improving insulin sensitivity (Liang and Koya, 2010; Lin et al., 2013) and c-peptide levels through effective treatment against metabolic disturbances such as hyperglycemia (Peplow and David, 2012; Tjipto, Saputra & Sutrisno, 2014), inducing weight loss (Cho et al., 2009; Tur et al., 2014; Firouzjaei et al., 2016) owing to its beneficial influence on hormones such as insulin, cholecystokinin, ghrelin and leptin (Zhang, et al. (2018), improving lipid metabolism (Liang and Koya, 2010; Lin et al., 2013; Firouzjaei et al., 2016) while capable of reducing inflammation (Wei et al., 2015). Additionally, acupuncture is able to control co-morbidity conditions such as anxiety (Frisk et al., 2012; David et al., 2013), depression (David et al., 2013), pain (Manyanga et al., 2014; Helianthi et al., 2016), stress (Mishra et al., 2017), sleeping disorder (Cao et al., 2009; Dong et al., 2017) and hence improved quality of life (Frisk, 2012; Vinjamury, 2013; Xiong et al., 2016).

The United States National Institutes of Health (NIH) has also acknowledged that acupuncture can be used as adjunctive treatment for diabetes (Takahashi, 2009). Equally, systematic reviews have also affirmed positive results of acupuncture as a complementary therapy for T2DM (Cai and Peng, 2010; Chen et al., 2019), diabetes complications (Yang, 2013; Lin et al., 2013; Cheok, 2013; Liu, 2016; Xiong, 2016) and obesity (Cho et al., 2009; Zhang et al., 2017; Zhong et al., 2020). Study showed that acupuncture was more effective than mecobalamin (RR 1.31, 95%CI 1.21-1.42), vitamin B1 and B12 (RR 1.55, 95% CI 1.33-1.80), and no treatment (RR 1.56, 95% CI 1.31-1.85) on global neuropathic symptoms (Chen et al., 2013). Additionally, acupuncture was highly effective in decreasing body mass index (BMI)(MD=-1.22, 95%CI=-1.87 to -0.56; P<0.001) and body weight (MD=-1.54, 95% CI=-2.98 to -0.11; P<0.001) when compared to sham acupuncture in reducing obesity (Zhang, et al. 2018). However, multiple methodological flaws with substantial risk of bias have been identified in many of these RCTs (Bo et al., 2012; Yang, 2013; Xiong et al., 2016; Dimitrova et al., 2017; Zhong et al., 2020).

Safety is one of the most important issues in the use of acupuncture in clinical practice, especially for diabetes mellitus. According to various prospective studies in China, Korea, Japan, Germany and the United Kingdom, serious adverse event associated with acupuncture is rare (Park et al., 2010; Witt et al., 2011; He et al., 2012; Birch, 2013) where the incidence rate was about 0.004%. Moreover, systematic reviews on acupuncture published between January 2005 until March 2013 found that adverse events were not significant and suggested for more standardized procedures including adverse events reporting mechanisms, safe practice procedures, acupuncturist’s qualifications and training (Hempel, Taylor, Solloway et al., 2013). Nevertheless, as compared with medications routinely prescribed in primary care, acupuncture is a relatively safe treatment modality with minimum side effects if it is performed properly by a well-trained practitioner (MacPherson et al., 2008).

Acupuncture is a new treatment modality, emerging within the last two decades. Currently, there are limited well-designed RCTs to determine the efficacy of acupuncture on diabetes mellitus. The purpose of this study is to conduct a clinical trial to assess the effect of acupuncture as adjunctive therapy in patients with T2DM in accordance with the consolidated Standards for Reporting of Trials Statement 2010 (CONSORT 2010)(Schulz Altman & Moher, 2010) and revised Standards for Reporting Interventions Controlled Trials of Acupuncture 2010 (STRICTA 2010)( MacPherson et. al., 2010) guidelines.

## 1. 2 Objectives:

### 1.2.1 General objective:

To determine the effect of acupuncture treatments on Homeostasis model assessment-insulin resistance (HOMA-IR) and health-related quality of life (HRQoL) in patients with T2DM***.***

### 1.2.2 Specific Objectives:

1. To compare
2. Socio-demographic background (age, gender, ethnicity, marital status, levels of education, employment, personal income);
3. Clinical characteristics (duration of disease, glycemic control, family history of disease)
4. Physical examinations (body weight, body mass index (BMI) and waist circumference (WC)

between acupuncture and control groups at baseline.

1. To examine the effect of acupuncture towards change in the HOMA-IR and/or HRQoL within and between acupuncture and control groups after treatment.
2. To identify the presence of adverse event in patients with T2DM between acupuncture and control groups.

## 1.3 Hypothesis:

There is significant effect of acupuncture towards change in the HOMA-IR and/or HRQoL within and between acupuncture and control groups after treatment.

## 1.4 Research Framework

This study intended to investigate the effect of acupuncture treatments on HOMA-IR and HRQoL of T2DM patients in Malaysia context. The effectiveness of the intervention will be examined through the change in HOMA-IR and/or HRQoL within and between the acupuncture and control groups after treatment. Consequently, the safety of acupuncture treatment will be identified between the acupuncture and control groups. A total of 10 sessions of acupuncture or placebo treatment will be provided to the intervention and control groups. Both groups will continue with their routine diabetes care. The primary outcome is the change of HOMA-IR while the secondary outcome is the change in HRQoL among two intervention groups. Figure 1 shows the treatments and variables to be investigated in this study.

**
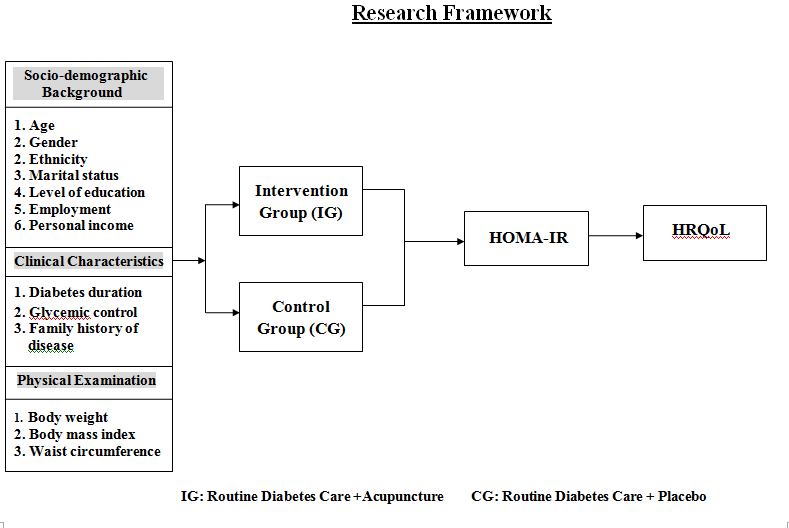
**

**Figure1:** Research Framework

# 2. Methodology

## 2.1 Study Location

The study will be conducted at seven centres in Malaysia namely i) Hospital Pengajar Universiti Putra Malaysia (HPUPM), Persiaran Mardi, 43400 Serdang, Selangor DE; ii) University Malaya Medical Centre, Jalan Universiti, 59100 Kuala Lumpur, Selangor DE; iii) TCM Clinical Skills Training Centre, School of Traditional Chinese Medicine, Xiamen University Malaysia (XMUM), Jalan Sunsuria, Taman Sunsuria, 43900 Sepang, Selangor DE; iv) Klinik Kesihatan Bukit Kuda, Jalan Batu Tiga Lama, 41300 Klang, Selangor DE; v) Klinik Kesihatan Bandar Botanik, Tingkat 1, Blok A, Jalan Langat, 41200 Klang, Selangor DE; vi) Pusat Kesihatan Universiti Putra Malaysia, Persiaran Mardi, 43400 Serdang, Selangor DE and vii) Setia Chinese Medical and Acupuncture Centre, Trefoil @ Setia City, No. 2, Jalan Setia Dagang AH U13/AH, 40170 Shah Alam, Selangor DE. Permission will be obtained from the respective hospitals/institutions before conducting the research. The first three centres are either public or private university teaching clinics, the fourth, fifth and sixth centres are public or university health clinics while the last centre is a private clinic. Among all the centres, the third and last centres are providing TCM medical services to the public. The involvement of XMUM in the study in relation to research assistance are a) use of TCM Clinical Skill Training Centre, School of Traditional Chinese Medicine as one of the study site; b) assign qualified and experienced volunteer acupuncturist(s) for the study and c) assist and coordinate the research at XMUM site.

## 2.2 Study Design

This is a randomized, double-blind (patients and practitioner), placebo-controlled and parallel design trial. The reporting of the trial will adhere to the CONSORT (Schulz, Altman & Moher, 2010) and STRICRTA (MacPherson et. al., 2010) recommendations.

## 2.3 Sampling Frame

The current study focuses on all the states in Malaysia in order to facilitate the patient recruitment process. As such, all diabetes patients in Malaysia will serve as sampling frame and those who fulfill inclusion criteria of the study will be included.

## 2.4 Sample Size

The sample size calculation is based on previous study (Firouzjaei et al. 2016) where the mean differences of HOMA-IR and standard deviation between two groups were 1.23 and 1.25 respectively. For the mean comparison method of two-sample t-test model, a significance level of 5% z_(1-∂/2)_ = 1.96 and a power of 90% z_(1-ß)_ = 1.28 will be utilized to calculate the required sample size.

**2 x (Z_∂_ + Z _ß_)^2^ x** $\boldsymbol{\sigma}$**^2^ (Noordzij et al., 2010)**

n **= -----------------------**

$\boldsymbol{\delta}$ **^2^**

**2 x (1.96 +** 1.**28)^2^ x (1.25)^2^**

**= -------------------------------- = 22**

**(**1.23**)^2^**

For equal allocation of the two groups, the total sample size required when considering a high dropout rate of 35*%* shall be 60 participants, with 30 participants in each group.

## 2.5 Study Population

Subjects will be recruited through government or private medical centres, community referral and mass media advertising based on criteria below:

Inclusion criteria**:**

1. Malaysian, age between 30 - 69 years old of either gender and with BMI ≤ 40.0 kg/m²
2. T2DM patients with fasting venous plasma glucose FPG ≥ 7.0 mmol/L (126 mg/dL) and glycated hemoglobin (HbA1c) ≥ 6.5% (Clinical Practice Guidelines [CPG], 2015; WHO, 2016; ADA, 2017)
3. Individuals have had T2DM more than one year
4. Diabetes under oral anti-diabetic agents on a stable dose over the previous 3 months and not under insulin therapy

Exclusion criteria**:**

1. Individuals with acute or chronic health problems (eg. chronic kidney disease [stage 4 and 5]/heart/liver failure, cancer, cardiovascular disease, stroke, physical disability, mental illness, nephrotic syndrome, decompensated congestive heart failure and oedema on the abdomen)
2. Needle phobia or allergy to adhesive plaster
3. Planning to move out from Malaysia within 4 months’
4. Being pregnant, planning for pregnancy or lactating women

## 2.6 Randomization and allocation strategy

Simple, complete randomization is performed using a randomization software (Stat Trek’s random number generator-http://stattrek.com/statistics/random-number-generator.aspx). Eligible participants are randomized into either acupuncture or placebo group in a 1:1 allocation ratio. The permuted block randomization will be utilized sequentially to minimize between-group differences on age, duration of diabetes mellitus and HOMA-IR level. The press needles and placebos are identical in appearance. They are repacked in identical plastic bags and consecutively numbered for each subject according to the randomization schedule. Each subject is assigned an order number. The plastic bags will be distributed to the principle investigator at the sites (Ng, Jeya or Cheok) and practitioner(s) will receive the corresponding repacked plastic bags which contain either press needles or placebos. Randomization sequence and allocation will be concealed to all researchers, subjects, and acupuncturists and laboratory personnel. Sequentially numbered, sealed opaque envelopes will be used to conceal treatment procedures. The randomization sequence will remain concealed until the end of the study.

## 2.7 Interventions

Subjects will receive ten sessions of real or placebo acupuncture treatment using press needle (PYONEX; 0.2 mm x 1.5 mm made by SEIRIN Corporation) over six weeks. Treatments will be administered by 3 qualified TCM practitioners or acupuncturists. They are required to undergo an intensive training to ensure consistency of the administered intervention. A standard operation procedure will be provided during the training such as identification of the acupoint location, compulsory acupoints to be used in each session, technique in performing the study treatment and the time to press needles after insertion of the needles. They will also be briefed about risk management of the trial during the training. To ensure the practitioners are masked to the group allocation, practitioners are requested not to check for the presence of a sharp tip below the plaster during the intervention***.***

Treatments will be performed twice in a week; however, a once-a-week treatment to a three-times-a-week treatment is permitted, but ten treatments must be completed within six weeks. After all the Pyonex press needles are fixed, acupuncture points will be pressed for at least three times (at 0, 15 and 30 minutes) for about 30 seconds, with the degree that can be tolerated by the subject. The follow-up for any adverse event occurs will be 1 week and the total study period will be 7 weeks. For those who are under lifestyle modification or drug treatment during intervention, they are informed to continue with their existing treatment regimens. Any medication and doses received by the participants will be recorded and monitored during the course of the study to ensure the medications are maintained throughout the study. Subjects are also advised to notify the acupuncturist immediately if there are any changes in their current treatments/circumstances such as personal details, medication or get pregnant (for woman) during the study. Safety or adverse event in each session will also be recorded.

### 2.7.1 Treatment group

The treatment group will receive manual active acupuncture treatments. A standardized treatment with 10 needles at 10 acupuncture points on abdomen area such as Zhongwan (Ren-12), Xiawan (Ren-10), Qihai (Ren-6), Guanyuan (Ren-4), Tian Shu (St-25), Daheng (Sp-15), Shangqu, (M-HN), Jinhe (M-HN)/Qixue (Kd-13), Shuidao (St-28) and Liang Men (St-21) are selected based on literature reviews and recommendation by international experts (Yang and Liu, 2015; Kim et al., 2015; Zhong et al., 2016; Firouzjaei et al., 2016). Modification is allowed, particularly less points according to subject’s symptom during treatment. The number and name of acupuncture points used in each session will be documented. The details of acupuncture points are listed in Table 1.

**Table 1:** Abdominal acupuncture points selected in the trial

| No. | Acupoint | Location |
| --- | --- | --- |
| 1 | Zhongwan (Ren-12) | On the anterior midline, 4 cun above the umbilicus |
| 2 | Xiawan (Ren-10) | On the anterior midline, 2 cun above the umbilicus |
| 3 | Qihai (Ren-6) | On the anterior midline, 1.5 cun below the umbilicus |
| 4 | Guanyuan (Ren-4) | On the anterior midline, 3 cun below the umbilicus |
| 5 | Tian Shu (St-25) | 2 cun lateral to the center of the umbilicus |
| 6 | Daheng (SP-15) | 3.5 cun lateral to the center of the umbilicus |
| 7 | Shangqu | 2 cun above the umbilicus (Ren-10/Xiawan), 0.5 cun lateral to the anterior midline |
| 8 | Jinhe (M-HN) or | 1.5 cun below the umbilicus, 0.5 cun lateral to the anterior midline (Ren-6/Qihai) |
|  | Qixue (Kd-13) | 3 cun below the umbilicus, 0.5 cun lateral to the anterior midline (Ren-4/Guanyuan) |
| 9 | Shuidao (St-28) | 3 cun below umbilicus, 2 cun lateral to anterior midline (Ren-4/Guanyuan) |
| 10 | Liang Men (St-21) | 4 cun above the umbilicus, 2 cun lateral (left) to the midline (Ren-12/Zhongwan) |

All subjects are required to take supine position during the treatments. New, disposable, sterilized, stainless-steel press needles will be utilized and inserted according to the acupuncture point selection sequence. The acupuncture points are disinfected with a 70% isopropyl alcohol cotton. In this study, an irradiating needling sensation (*de qi*) does not have to be achieved. “De qi” is a tingling, numbness, and heaviness feelings that occur after an acupuncture needle has been inserted in the body (National Cancer Institute [NCI], 2017). Unnecessary talking in the treatment room is also avoided***.*** During the 45 minutes needle retention period, subject’s abdomen will be covered to prevent exposure. If the subjects are not fit on that day, they will not be given any treatment and will be requested to come on other day. Similarly, if subject reports discomfort or encounter any adverse effect during intervention, acupuncture therapy will be discontinued and the treatment for that particular session will not be included.

### 2.7.2 Control group

The same procedure and acupuncture points are used for the control group. However, subjects are given placebo which is identical to the press needle except the needle element has been removed and replaced with a blunt knob in all aspects are indistinguishable to the press needles (Figure 2; Fleckenstein, Baeumler, Gurschler et al., 2014).

*
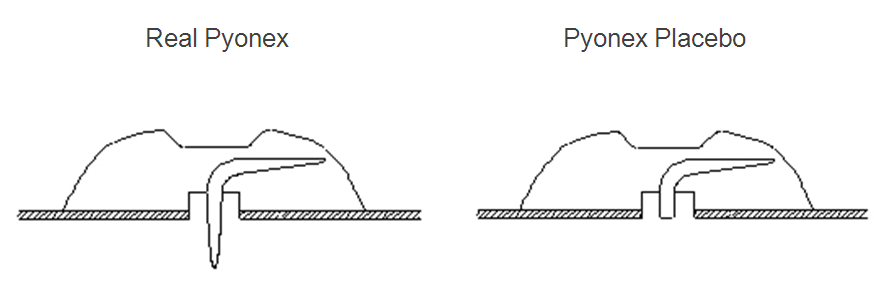
*

Figure 2: Real pyonex and pyonex placebo

The press needles and placebos are administered (inserted) according to the product directions and following same procedures (Miyazaki et al., 2009)***.*** The validity and credibility of the device had been well demonstrated in other studies (Fleckenstein, Baeumler, Gurschler et al., 2014; Bai et al., 2015; Schroder et al., 2017). To ensure the practitioner is masked to the group allocation, acupuncturist is requested not to check for the presence of a sharp tip below the plaster during the intervention***.*** Figure 3 demonstrated the schematic diagram of study design.


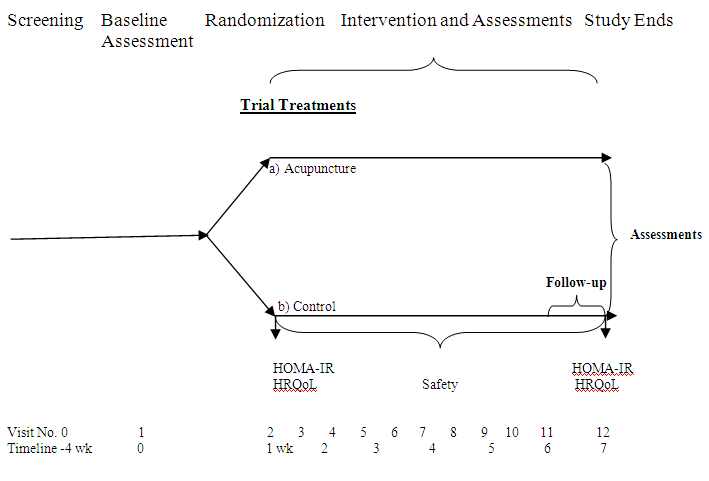


**Figure 3:** Schematic diagram of study design

## 2.8 Study Approval

The study is registered with National Medical Research Register (NMRR), Ministry of Health [NMRR-18-2412-43863] and approved by the Ethics Committee for Research Involving Human Participants (JKEUPM) of Universiti Putra Malaysia (JKEUPM-2018-294). Subjects will be informed of the purpose, procedures and safety of the trial and are free to leave without obligation. Subjects in this study are free to make informed choices in taking part. Written informed consent will be obtained from all the subjects’ prior enrollment of the study

## 2.9 Study Procedure

The procedure of this study is shown in Figure 4.

**
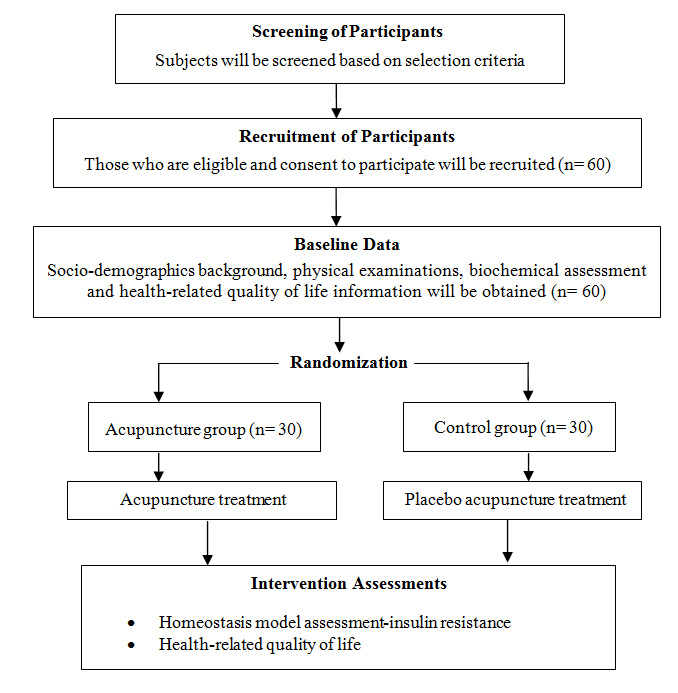
 Figure 4: Flow chart of study procedure**

Upon obtaining ethical approval for this study, subjects with T2DM are invited to a screening session. A self-developed screening questionnaire will be administered to identify subjects’ eligibility (Appendix A). T2DM will be confirmed with subject’s HbA1c ≥ 6.5% and morning FPG ≥ 7.0 mmol/L (126 mg/dL) (CPG, 2015; WHO, 2016; ADA, 2017) based on subjects’ diabetes book, medications packages, and clinic follows up cards. Those fulfill all inclusion criteria and granting written informed consent will be recruited (Appendix B). Subjects who are taking part in this study will not have to pay for participating. Similarly, no payment will be provided for their participation in this trial.

## 2.10 Measurements

Measurements are performed at baseline and week 7 of the study. A case record form (CRF) will be developed to ascertain socio-demographics, clinical characteristics, physical examinations and HRQoL information of the participants (Appendix C).

### 2.10.1 Socio-demographics and clinical characteristics

Information such as age, gender, ethnicity, marital status, educational level, personal monthly income, duration of diabetes mellitus, and family history of diabetes mellitus will be self-reported from subjects.

### 2.10.2 Body weight and height

Subjects will be measured for weight in kilogram to the nearest 0.1 kilogram, using a digital scale TANITA model HD-382 weighing machine in a standing position. After each measurement, the weighing machine will be reset to zero. The respondent will be requested to stand on the middle of the weighing machine, with the head looking straight forward, arms by the side with minimal clothed and without shoes.

Height will be measured using SECA body meter model 206 in a standing position without shoes while shoulders are in a normal state using a body meter attached to a rigid wall. The accuracy of this device is 0.05 centimeter. Two measurements will be used for both weight and height and the average of the two values will be used in the analysis. Body mass index (BMI) is calculated as weight in kilogram divided by height in meters squared (BMI=Weight(kg)/Height^2^(m²).

### 2.10.3 Waist Circumference

###

Waist circumference (WC) will be measured using SECA measuring tape to the nearest 0.05 centimeter. Respondent will be requested to stand erect and has relaxed the abdominal muscles. Locate the top of the hip bone (iliac crest) and measure at a level just above the iliac crest, and positioning the tape horizontally. Measurements are taken at the end of normal expiration. Two measurements will be taken and average of the two values will be used for the analysis. Central obesity for Asian population is defined as having WC ≥ 90cm for men and ≥ 80cm for women based on WHO/IASO/IOTF criteria (WHO, 2000). All the above physical measurements such as height and weight, waist circumference will be carried out by researcher.

### 2.10.4 Biochemical Assessment

Samples of fasting morning blood (fasting at least 10 hours) will be collected from each subject by qualified personnel for measuring Homeostasis model assessment–insulin resistance (HOMA-IR). HOMA is a method used to quantify insulin resistance and ß-cell function from basal (fasting) glucose and insulin or C-peptide concentrations. It has been proved to be a robust clinical and epidemiological tool for the assessment of insulin resistance (Mathews et al., 1985).

A total of 10 ml/L or approximately 2 teaspoons of venous blood from cubital vein will be performed using venipuncture method. The blood samples will be analysis at pathology laboratory centre for fasting insulin levels, fasting glucose using heparin tubes and sodium fluoride tubes respectively. In this study, fasting insulin and glucose concentrations will be used to derive the HOMA-IR [fasting serum insulin (μU/ml)×fasting plasma glucose (mmol l-1)/22.5] (Mathews et al., 1985) and blood samples will be disposed in four weeks’.

### 2.10.5 Health-related Quality of Life

###

Patient’s health-related quality of life will be assessed using a validated World Health Organization Quality of Life Assessment: Brief Version (WHOQOL-BREF)(The WHOQOL Group, 1998; Hasanah, 2003). It consists of 26 items which represent four domains of psychological, social relationship, environmental, physical health domains and two facets of overall quality of life and general health. The question scores of all 26 items ranged from 1 to 5.

WHOQOL-BREF has been found to be comparable to the WHOQOL-100 and other WHOQOL (The WHOQOL Group, 1998; Hasanah, 2003). It is able to discriminate between patients and healthy people in revealing characteristic impairment of certain illness, including diabetes (Hasanah, 2003). The English or Malay versions of this instrument have been validated in Malaysia (Hasanah, 2003; Chew, Mohd-Sidek & Sharif-Ghazali, 2015). The Cronbach’s alphas coefficient of the WHOQOL-BREF was found to be satisfactory, ranging from 0.66 to 0.84 (WHO, 1998; Hasanah, 2003; Chew, Mohd-Sidek & Sharif-Ghazali, 2015).

### 2.10.6 Safety

###

Safety or adverse event suspected related to the treatment, through the symptoms reported by the patients, and observations by researcher at every visit will be collected. Adverse event is defined as any unfavorable and unintended sign, symptom, or disease temporally associated with the use of a medical treatment or procedure that may or may not be considered related to the medical treatment or procedure. It will be recorded on case record form according to terms of intensity: grade 1- mild; asymptomatic or mild symptoms; clinical or diagnostic observations only; intervention not indicated; grade 2- moderate; minimal, local or noninvasive intervention indicated; grade 3- severe or medically significant but not immediately life-threatening; hospitalization or prolongation of hospitalization indicated; disabling; grade 4- life-threatening consequences; urgent intervention indicated and grade 5- death related to adverse event (NCI, 2017)**.** Nonetheless, the patients and institution will be covered by insurance or indemnify against claims arising from the trial.

# 3. Early termination, drop-outs, withdrawal and decoding procedure

Early termination of the trial is allowed if the risks are found to outweigh the potential benefits, if there is any serious adverse event that cause by the trial or when there is conclusive proof of positive and beneficial results. In the event that subject does not turn up for treatment or assessment, they are invited to join the other subsequent session. Subject’s is considered drop-outs if they have missed their appointments for more than three times, they do not attend the baseline or assessment session (cause missing data), they become pregnant (for women), withdrawal of his/her own decision or advised by the researcher (for example increase 20% doses of diabetes medication or using acupuncture at other clinic during intervention). Additionally, withdrawal subject will be followed up until the end of the study and every effort must be made to report the observation. Last but not least, decoding procedure shall undertake if there is any serious adverse event occurred.

# 4. Data Collection

The research will be undertaken from 2021 until 2022. Subject’s socio-demographics and clinical characteristics are obtained during baseline visit. HOMA-IR, HRQoL, body weight, height, BMI and WC will be collected at baseline and at week 7 after intervention. However, safety information will be collected at every session and assessed at week 7 after the trial.

# 5. Statistical Analysis

Data will be analyzed using SPSS Statistics 26.0 (IBM Corp., New York, USA). Data will be presented as mean ± SD for continuous parameters and percentage for categorical data. Kolmogoro-Smirnov tests for normality will be performed on each parameter before analysis. Comparisons of background and baseline continuous variables between intervention and control group will be examined using independent t-test, while for categorical variables the chi-square test will be used. Intention-to-treat analysis (ITT) will be performed, and all patients enrolled will be included in the analysis, irrespective of compliance. The missing data is imputed using last observation carried forward method. Paired t-test will be carried out in analyzing the statistical significance of mean pre-post change in the parameters. The changes between groups on two time-points (pre-post) will be conducted using analysis of covariance (ANCOVA) if covariates are present. A repeated measure ANCOVA is performed to compare changes between groups at baseline, week 3/4 (6^th^ visit) and week 7 of intervention while controlling for a confounding variable. Mc Nemar’s test is utilized for matched pair’s comparison. However, if data are not normally distributed, non-parametric tests are used. The significant level for all tests is set at p<0.05 (two-tailed).

# 6. Expected Outcome

Based on previous studies of meta-analyses and systematic reviews, it is expected that results will be in favour for acupuncture than control group. Subjects in the intervention group will be expected to have some improvements in their HOMA-IR levels and HRQoL as compared to the control group.

# 7. Time schedule

A breakdown of the specific tasks to be accomplished within timeframe can be seen in the table below. See details of Carta Gantt as follows:

| Procedure | Sep 2015 -  Feb 2017 | Sep 2017 - Feb  2018 | Sep 2018 | Feb  2019-  Feb  2020 | Sep  2020 | Feb  2021 | Sep  2021 | Feb  2022 |
| --- | --- | --- | --- | --- | --- | --- | --- | --- |
| Literature search & supervision | ∕ | ∕ | ∕ | ∕ | ∕ | ∕ | ∕ | ∕ |
| Proposal preparation, presentation & approval | ∕ | ∕ | ∕ | ∕ | ∕ |  |  |  |
| Improving methodology & research design | ∕ | ∕ | ∕ | ∕ | ∕ | ∕ |  |  |
| Data collection |  |  |  |  | ∕ | ∕ | ∕ |  |
| Report writing & publication |  |  |  |  |  | ∕ | ∕ |  |
| Preparing first draft |  |  |  |  |  |  | ∕ |  |
| Preparing second draft |  |  |  |  |  |  | ∕ | ∕ |
| Finalize and editing final report |  |  |  |  |  |  |  | ∕ |
| Submission of thesis |  |  |  |  |  |  |  | ∕ |
| VIVA presentation |  |  |  |  |  |  |  | ∕ |

# 8. Conflict of interest

The authors have no conflict of interest.

# 9. References

American Diabetes Association. (2017). *Standards of Medical in diabetes -2017*. Vol.

40, Suppl. 1.

Bai, Y., Chen, S., Chen, L. and Wang, B. (2015).Postoperative pain of mixed

hemorrhoid treated by embedding needles in Erbai. World Journal of

Acupuncture-Moxibustion (WJAM). 25(1): 59-61.

Bassuk, SS, Manson, JE. Epidemiological evidence for the role of physical activity in

reducing risk of type 2 diabetes and cardiovascular disease. *J Appl Physiol*

(1985). 2005; 99(3): 1193–1204.

Birch, S., Alraek, T and Norheim, AJ. (2013). *The Journal of Alternative and*

*Complementary Medicine*. 19(10): 845-850. DOI: 10.1089/acm.2012.0639.

Bo, C, Xue, Z, Yi, G, Zelin, C, Yang, B, Zixu, W and Yajun, W. (2012). Assessing

the quality of reports about randomized controlled trials of acupuncture

treatment on diabetic peripheral neuropathy. *PLoS One*. 7(7): e38461.

British Acupuncture Council. Acupuncture. 2019.

https://www.acupuncture.org.uk/public-content/public-traditional-

acupuncture/3825-acupuncture.html

Cai and Peng (2010). Meta analysis on acupuncture treatment of diabetes. *Chinese*

*Archives of Traditional Chinese Medicine.* 28(11): 2412-2415.

Charles, MCJ. (1998). Oral therapy in type 2 diabetes: Pharmacological properties

and clinical use of currently available agents. *Diabetes Spectrum*. 11(4): 211-221.

Chen, W, Yang, GY, Liu, B, Manheimer, E, Liu, JP. (2013). Manual acupuncture for

treatment of diabetic peripheral neuropathy: a systematic review of randomized

controlled trials. *PLOS*. 8(9):e73764.

Cheok, YC. (2013). *Acupuncture for diabetic complications – a systematic review*

*and meta-analyses*. Unpublished master dissertation, University of Middlesex, United Kingdom.

Chew, B . H., Mohd-Sidik, S and Sharif-Ghazali, S. (2015). Negative effects of

diabetes-related distress on health-related quality of life: an evaluation among

the adult patients with type 2 diabetes mellitus in three primary healthcare

clinics in Malaysia. *Health and Quality of Life Outcomes*. 13: 187.

Cho, SH, Lee, JS, Thabane, l and Lee, J. (2009). ‘Acupuncture for obesity: a

Systematic review and meta-analysis’. *International Journal Obeseity*. 33(2):

183-96.

Cisneros, LDL., Teixeira, RLL and Navarro, TP. (2015). Quality of life of

people with diabetes mellitus pre revascularization surgery and post

amputation: an exploratory study. *Diabetology and Metabolic Syndrome.*

7(1):A19.

Clinical Practice Guidelines (CPG). (2015). *Management of type 2 diabetes mellitus,*

*5^th^ Edition*. Putrajaya: Malaysian Endocrine & Metabolic Society.

Currie, CJ, Peyrot, M, Morgan, CL et al. (2012). The impact of treatment

noncompliance on mortality in people with type 2 diabetes. *Diabetes Care*.

35:1279–1284. <http://dx.doi.org/10.2337/dc11-1277>.

David, P, Sniezek, DC, FAAMA and Imran J. Siddiqui. (2013). Acupuncture for

Treating Anxiety and Depression in Women: A Clinical Systematic Review.

*Medical Acupuncture*. 25(3): 164-172. doi: 10.1089/acu.2012.0900.

Department of Health: the management of adult diabetes services in the NHS. (2012). House of Commons Committee of Public Accounts. *Department of Health: the management of adult diabetes services in the NHS. Seventeenth report of session 2012-13*. London: The Stationery Office Limited.

Dimitrova A, Murchison C, Oken B. Acupuncture for the Treatment of Peripheral

Neuropathy: A Systematic Review and Meta-Analysis. J Altern Complement Med. 2017;23(3):164–179. doi:10.1089/acm.2016.0155.

Dong, B, Chen, Z, Yin, X et al. (2017). The efficacy of acupuncture for treating

depression-related insomnia compared with a control group: a systematic

review and meta-analysis. *BioMed Research International*. ID9614810,

http://doi.org/10.1155/2017/9614810.

Elisabet, S. (2008). Acupuncture in Polyscystic Ovary Syndrome: current experimental

and clinical evidence. *Journal of Neuroendocrinology*. 20: 290-298.

Evans, N. Acupuncture for anxiety. *CNS Neurosci Ther*. (2012). 18(4):277-84. doi:

10.1111/j.1755-5949.2011.00254.x. Epub 2011 Jun 7.

Feisul, MI, Azmi, S. (Eds). National Diabetes Registry Report, *vol. 1, 2009-2012*.

Kuala. Lumpur; Ministry of Health Malaysia; 2013.

Firouzjaei, A, Li. GC, Wang, N, Liu, WX and Zhu, BM. (2016). Comparative

evaluation of the therapeutic effect of metformin monotherapy with metformin and acupuncture combined therapy on weight loss and insulin sensitivity in diabetic patients. *Nutrition & Diabetes*. 6, e209; doi: 10.1038/nutd2016.16.

Fleckenstein, J., Baeumler, P. I., Gurschler, C., Weissenbacher, T., Simang, M. & et

al., (2014). Acupuncture for post anaesthetic recovery and postoperative

pain: study protocol for a randomized controlled trial. Trials. 15: 292.

Frisk, J, Kallstrom, AC, Wall, N, Fredrikson M and Hammar, M. (2012). Acupuncture

improves health-related quality-of-life (HRQoL) and sleep in women with

breast cancer and hot flushes. *Support Care Cancer*. 20(4):715-24.

doi: 10.1007/s00520-011-1134-8. Epub 2011 Apr 6.

**Gelaw, BK, Mohammed, A, Tegegne, GT. (2014). Nonadherence and Contributing**

**Factors among Ambulatory Patients with Antidiabetic. *Journal of Diabetes***

***Research.* Volume 2014, Article ID 617041, 9 pages.**

**http://dx.doi.org/10.1155/2014/617041**

Goedecke, JH, Ojuka, EO. (eds) (2014). Diabetes and Physical Activity. *Med Sport*

*Sci.* 60: 1-8. (DOI:10.1159/000357348)

Grant, RW., Pabon, L., Pandiscio, JC. Et al. (2011). Diabetes oral medication and

intensification: Patient views compared to current treatment guidelines. 37(1):

78-84. doi:10.1177/0145721710388427.

Hasanah, CI., Naing, L. and Rahman, ARA. (2003). World Health Organization

Quality of Life Assessment: Brief Version in Bahasa Malaysia. *Med J*

*Malaysia.* 58(1): 79-88.

Helianthi, DR, Simadibratai, C, Srilestari, A et al. (2016). Pain reduction after Laser

Acupuncture treatment in geriatric patients with knee osteoarthritis: a

randomized controlled trial. *Acta Med Indon-Indon J Intern Med.* 117-121.

Hempel, S., Taylor, S. L., Solloway, M., MIAKE-Lye, I. M., Beroes, J. M., Shanman,

R., Booth, M. J., Siroka, A. M., Shekelle, P. G. Evidence map of

acupuncture. VA-ESP Project #05-226; 2013.

He, WJ, Zhao, X, Li, YQ et al. (2012). Adverse events following acupuncture: A

systematic review of the Chinese literature for the years 1956–2010. *J Altern*

*Complement Med*. 18:892–901.

Holman, N, Young, RJ and Jeffcoate, WJ. (2012). Variation in the recorded

incidence of amputation of the lower limb in England. *Diabetologia.* 55:

1919-1925.

Ibrahim, R (2010). Diabetes mellitus type II: Review of oral treatment options. *International Journal of Pharmacy and Pharmaceutical Sciences*. 2(1): 21-30.

Institute for Public Health (IPH) 2015. *National Health and Morbidity Survey 2015*

*(NHMS 2015).* Vol. II: Non-Communicable Diseases, Risk Factors & Other

Health Problems; 2015.

International Diabetes Federation (IDF). (2015). *IDF Diabetes Atlas Seventh edition 2015*. [online].

www.oedg.at/pdf/1606_IDF_Atlas_2015_UK.pdf

International Diabetes Federation. *IDF Diabetes Atlas. 8th Edition* 8th edition.

Brussels: International Diabetes Federation, 2017.

James, PA, Oparil, S, Carter, BL et al. (2014). Evidence-based guideline for the

management of high blood pressure in adults report from the panel members

appointed to the Eight Joint National Committee (JNC 8). *JAMA.* 311(5): 507-

520.

Kassahun, A, Gashe, F, Mulisa, E and Rike, WA (2016). Nonadherence and factors

affecting adherence of diabetic patients to anti-diabetic medication in Assela

General Hospital, Oromia Region, Ethiopia. *J Pharm Bioallied Sci.* 8(2): 124-

129. doi: 10.4103/0975-7406.171696.

Khunti, S, Davies, MJ and Khunti, K (2015). Clinical inertia in the management of

type 2 diabetes mellitus: a focused literature review. *Br J Diabetes Vasc* *Dis.*

15(2): 65-69.

Kim, H. (2014). Analysis of variance (ANOVA) comparing means of more than two

groups. *Restorative Dentistry & Endodontics*, *7658*: 74–77.

Kim, T. K. (2015). T test as a parametric statistic. *Korean Journal of Anesthesiology*,

68(6): 540–546.

Kim, KW, Yoo, HH, Cho et al. (2015). Effects of acupuncture on serum metabolic

parameters in premenopausal obese women: study protocol for a randomized

controlled trial. *Trials*. 16: 327. DOI 10.1186/s13063-015-0867-y.

Letchuman, GR, Wan Bazaimoon, WM, Wan Mohamad, WB, Chandran, LR,

Tee, GH, Jamaiyah, H., Isa, MR, Zanariah, H, Fatanah, I. and Ahmad

Faudzi, Y. (2010). Prevalence of diabetes in the Malaysian National Health

Morbidity Survey III 2006. *Med J Malaysia.* 65(3): 180-6.

Liang, F and Koya, D. (2010). Acupuncture: is it effective for treatment of insulin

resistance. Diabetes, *Obesity and Metabolism*. 2(7): 555-569.

Lin, RT, Pai, HC, Lee, YC, Tzeng, CY, Chang, CH, Hung, PH, Chen, YI, Hsu,

TH, Tsai, CC, Lin, JG and Chang, SL (2013). Electroacupuncture and

rosiglitazone combined therapy as a means of treating insulin resistance and

type 2 diabetes mellitus: a randomized controlled trial. *Evidence-Based*

*Complementary and Alternative Medicine.* 2013:969824. doi:

10.1155/2013/969824.

Lin, RT, Tzeng, CY, Lee, YC, Chen, YI, Hsu, TH and Lin, JG and Chang, SL. (2014).

Acupoint-specific, frequency-dependent, and improved insulin sensitivity

hypoglycemic effect of electroacupuncture applied to drug-combined therapy

studied by a randomized control clinical trial. *Evidence-Based Complementary*

*and Alternative Medicine.* ID371475,

http://dx.doi.org/10.1155/2014/371475.

Liu, MJ, Liu ZC, Xu B, Zhang, W, Cai, JW. (2016). Review of systematic reviews and

Meta-analyses investigating Traditional Chinese Medicine treatment for type 2

diabetes mellitus. *Journal of Traditional Chinese Medicine*. 2016; 36(5): 555-

563.

MacPherson, H., Altman, DG, Hammerschlag, R., Li, Y, Wu, T, White, A, Moher,

D, on behalf of the STRICTA Revision Group. (2010). ‘Revised Standards

Reporting Interventions in Clinical Trials of Acupuncture (STRICTA):

Extending the CONSORT Statement’. *PLoS Medicine*. [online]. 7(6).

MacPherson, H., Hammerschlag, R., Lewith, G and Schnyer, R. (2008). *Acupuncture*

*Research. Strategies for Establishing an Evidence Base.* USA: Churchill Livingstone.

Matthews, DR, Hosker JP, Rudenski, AS, Naylor, BA, Treacher, DF, Turner, RC. (1985). Homeostasis model assessment: Insulin resistance and beta-cell function from fasting plasma glucose and insulin concentrations in man. *Diabetologia*. 28:412–9.

Miyazaki, S., Hagihara, A;, Kanda, R., Mukaino, Y. Nobutomo, K. (2009).

Applicability of press needles to a double-blind trial: a randomized, double-

blind, placebo-controlled trial. The Clinical Journal of Pain. 25(5):438-444.

DOI: 10.1097/AJP.0b013e318193a6e1

Morewitz, SJ. (2006). *Chronic diseases and health care: new trends in diabetes,*

*arthritis, osteoporosis, fibromyalgia, low back pain, cardiovascular disease,*

*and cancer.* New York: Springer.

NCI, National Cancer Institute, Common Terminology Criteria for Adverse Events (CTCAE) v5.0. NIH. 2017.

Noordzij, M, Tripepi, G. Dekke, FW, Zoccali, C, Tanck, MW, Jager, KJ. (2010). Sample size calculations: basic principles and common pitfalls. *Nephrol Dial Transplant.* 25: 1388-1393.

Olokoba, Obateru & Olokoba, (2012). Type 2 diabetes mellitus: a review of current trends. Oman Medical Journal. 27(4): 289-273. DOI 10. 5001/omj.2012.68

Oliva, J. Fernandez, A and Hidalgo, A. (2012). Health-related quality of life in diabetic people with different vascular risk. *BMC Public Health*. 12: 812

Park, JI, Lee, MS, Choi, JY et al. (2010). Adverse events associated with acupuncture:

A prospective survey. *J Altern Complement Med*. 16:959–963.

Peplow, PV and David, BG. (2012). Electroacupuncture for control of blood glucose

in diabetes: literature review. *Journal Acupuncture Meridian Study*. 5(1):1-10.

Podbielska, M, Banik, NL, Kurowska, E and Hogan, EL. (2013). Myelin Recovery in

Multiple Sclerosis: the challenge of Remyelination. *Brain Sci. 3*: 1282-1324.

doi:10.3390/brainsci3031282

Reach, G. Patients’ nonadherence and doctors’ clinical inertia: two faces of medical

irrationality. *Diabetes Manag*. 2015; 5(3): 167-181.

Ruzaidi, A, Abbe, MA, Nawalyah, AG, Muhajir, H, Pauliena, MB and Muskinah,

MS. (2008). Hypoglycaemic Properties of Malaysia Cocoa (Theobroma

Cocoa) Polyphenols-Rich Extract. *International Food Research Journal*.

15(3): 1-21.

Schroder, S., Meyer-Hamme, G., Friedemann, T., Kirch, S., Hauck, M., Plaetke, R.,

Friedrichs, S., Gulati, A. & Briem, D. (2017). Immediate pain relief in

adhesive capsulitis by acupuncture- a randomized controlled double-blinded

study. Pain Medicine. 2235-2247. Doi: 10.1093/pmm/pnx052.

Schulz, KF, Altman, DG, Moher, D, for the CONSORT Group. (2010).

‘CONSORT 2010 Statement: updated guidelines for reporting parallel group

randomised trials’. BMJ. [online]. 340, (c332).

Takahashi, H. (2009). Effects of Acupuncture on Terminal Cancer Patients in the

Home Care Setting. *Medical Acupuncture.* 21(2): 123-129.

The WHOQOL Group. (1998). Development of the World Health Organization

WHOQOL-BREF Quality of Life Assessment. *Psychological Medicine*. 28:

551-58.

Tjipto BW, Saputra K, Sutrisno TC. (2014). Effectiveness of acupuncture as an

adjunctive for diabetes mellitus: A randomized controlled trial. *Medical*

*Acupuncture.* 26 (6): 341-345.

Tur, FC., E, Kilic, TY and Temizyurek, Z. (2014). Therapeutic effects of acupuncture

on obesity and HbA1c. *European Journal of Integrative Medicine*. 7: 88-93.

Ummi, NY, Diana, M, Azahadi, O et al. Burden of premature mortality in Malaysia.

*Int J Public Health Res* 2013; 3: 249-56.

VanderPloeg, K., & Yi, X. (2009). Acupuncture in Modern Society . Journal of Acupuncture and Meridian Studies , 2(1), 26-33. doi:https://doi.org/10.1016/S2005-2901(09)60012-1

Vincent, C. (2001). The safety of acupuncture: acupuncture is safe in the hands of

competent practitioners. *BMJ*. 323:467–8.

Vinjamury, PS, Li, JZ, Hsiao, E, Huang, C., Hawk, C, Miller, J and Huang, Y. (2013).

Effects of acupuncture for cancer pain and quality of life – a case series.

*BioMed Central*. 8: 15.

Wei, Y, Dong, M, Zhang, H et al. (2015). Acupuncture attenuated inflammation and

inhibited Th17 and treg activity in experimental asthma. *Evidence-Based*

*Complementary and Alternative Medicine*. Article ID 340126.

http://dx.doi.org/10.1155/2015/340126

White, A. (2004). A cumulative review of the range and incidence of significant

adverse events associated with acupuncture. *Acupunct Med*. 22(3): 122-133.

Witt, CM, Pach, D, Reinhold, T, et al. (2011). Treatment of the adverse effects from

acupuncture and their economic impact: A prospective study in 73,406 patients

with low back or neck pain. *Eur J Pain*. 15:193–197.

World Health Organization (2019). *Classification of diabetes mellitus 2019*.

Licence: CC BY-NC-SA 3.0 IGO

World Health Organization. (2000). *The Asia-Pacific perspective: redefining obesity*

*and its treatment*. Health Communications Australia, 55p.

World Health Organization (2016). *Global report of diabetes*.

http://apps.who.int/iris/bitstream/10665/204871/1/9789241565257_eng.pdf

World Health Organization. (2013). *Physical Inactivity: A Global Public Health*

*Problem*.

http://www.who.int/dietphysicalactivity/factsheet_inactivity/en/index.html

World Health Organization's Quality of Life group: WHOQOL-BREF Introduction,

Administration and Scoring (1998), Field Trial version.

World Health Organization. (2013). *The* *WHO Traditional Medicine (TM)*

*Strategy 2014–2023.*

[www.who.int/about/licensing/copyright_form/en/index.html](http://www.who.int/about/licensing/copyright_form/en/index.html).

Xiong, WJ, Feng, X, Liu, JP, Chen, W. (2016). Electroacupuncture for treatment of

diabetic peripheral neuropathy: a systematic review of randomized controlled

trials. *Journal of Traditional Chinese Medical Sciences*. 3:9-21.

Yang, MX, Li, XM, Liu, SH. (2013). Meta-analysis of acupuncture for relieving non-

organic dyspeptic symptoms suggestive of diabetic gastroparesis. *BMC*

complementary and alternative medicine. 13(1): 311.

Yang, Y, Liu, Y. (2015). BO's abdominal acupuncture for obese type-2 diabetes

mellitus. *Zhongguozhenjiu = Chinese acupuncture & moxibustion*. 354: 330-

334.

Zafar, A, Davies, M, Azhar, A, Khunti, K. (2010). Clinical inertia in management of

T2DM. *Primary* *Care* *Diabetes*. 4(4): 203-207.

http://dx.doi.org/10.1016/j.pcd.2010.07.003.

Zanariah, H, Sri WT, Harvinder, K. and Winnie Chee, SS. (2015). Diabetes Care in

Malaysia: Problem, New models, and Solution. *Annals of Global Health.*

81(6):851-862.

Zhang, K., Zhou, S., Wang, C., Xu, H. & Zhang, L. (2018). Acupuncture on obesity:

clinical evidence and possible neuroendocrine mechanisms. *Evidence-Based*

*Complementary and Alternative Medicine.* (6409389: 1-15.

https://doi.org/10.1155/2018/6409389N

Zhong, LD, Kun, W, Lam, TF et al. (2016). The combination effects of body

acupuncture and auricular acupressure compared to sham acupuncture for

body weight control: study protocol for a randomized controlled trial. *Trials.*

17: 346. doi: 10.1186/S13063-016-1458-2.

Zhong YM, Luo XC, Chen Y, et al. Acupuncture versus sham acupuncture for simple

obesity: a systematic review and meta-analysis. *Postgrad Med J*.

2020;96(1134):221‐227. doi:10.1136/postgradmedj-2020-137221.
