# Supplementary material for: Effectiveness of acupuncture as adjunctive therapy in type 2 diabetic: Study protocol for a randomized controlled trial
Source: PLoS One. 2023 Sep 20;18(9):e0284337. doi: 10.1371/journal.pone.0284337 (PMC10511073; doi:10.1371/journal.pone.0284337)
Supplement: S1 Checklist — (DOCX) [file pone.0284337.s005.docx]

**SPIRIT 2013 checklist**

| Section/item | Item  No | Description |
| --- | --- | --- |
| Administrative information | | |
| Titles | 1 | A randomized, multi-center, investigator and patient-blinded, 6 weeks, parallel-group study protocol to compare the efficacy of acupuncture versus placebo group as adjunctive therapy in type 2 diabetic. |
| Trial registration | 2a | UTN: U1111-1219-3545  ClinicalTrials.gov identifier: NCT04829045  Institute Approval Letter: JKEUPM-2018-294 |
|  | 2b | See S1 Appendix |
| Protocol version | 3 | Issue date: 11 July 2021  Authors: Cheok Yean Chin, Zalilah Mohd Shariff, Chan Yoke Mun, Lee Ping Yein and Ng Ooi Chuan |
| Funding | 4 | Self-funding |
| Roles and  responsibilities | 5a | Study: Cheok Yean Chin conceived of the study. Zalilah Mohd Shariff, Chan Yoke Mun and Lee Ping Yein initiated the study design. Cheok Yean Chin, Zalilah Mohd Shariff, Chan Yoke Mun and Ng Ooi Chuan contributed to study implementation. Manuscript: Cheok Yean Chin prepared original draft; Zalilah Mohd Shariff reviewed and finalized the manuscript. Chan Yoke Mun, Lee Ping Yein, and Ng Ooi Chuan read and approved the final version of manuscript. |
|  | 5b | The author(s) received no specific funding for this work. |
|  | 5c | Not relevant |
|  | 5d | Cheok Yean Chin, Zalilah Mohd Shariff, Chan Yoke Mun and Lee Ping Yein are responsible for the preparation of the study protocol, case record form (CRF) and relevant documents with Ng Ooi Chuan provided inputs for revision. All investigators will oversee the trial implementation. Cheok Yean Chin and Ng Ooi Chuan will assist in recruiting patients, performing, and overseeing treatment at site. All investigators will contribute to any report/publication outcomes of this trial. |
| **Introduction** | | |
| Background and rationale | 6a | Introduction: Globally, the number of people with diabetes mellitus is increasing with most of them have type 2 diabetes mellitus.  Mechanism: Type 2 diabetes mellitus involves a decline in b-cell function and increase in insulin resistance which are associated with many health complications. Among the several mechanism-based drugs available for treatment of type 2 diabetes mellitus, only insulin sensitizers have direct effects on insulin resistance such as biguanides and thiazolidinediones. However, pharmacological agents have limitations which may be associated with the fear of side-effects of the medications.  Existing knowledge: Several randomized controlled trials have confirmed the effectiveness of acupuncture on type 2 diabetes mellitus and its complications. Systematic reviews have also affirmed the benefits of acupuncture in patients with diabetes mellitus Acupuncture is able to control many co-morbidity conditions of diabetes mellitus and hence improves quality of life.  Need for a trial: Due to reported trials appear to have multiple methodological flaws, this double-blind, placebo controlled clinical trial is designed to investigate the effect of acupuncture as adjuvant therapy in patients with type 2 diabetes mellitus in accordance with the consolidated Standards for Reporting of Trials Statement 2010 (CONSORT 2010)( Schulz, Altman & Moher et al., 2010) and revised Standards for Reporting Interventions Controlled Trials of Acupuncture 2010 (STRICTA 2010)(MacPherson et al., 2010) recommendation. Any adverse events occurring will be evaluated according to National Cancer Institute- Common Terminology Criteria for Adverse Events guidelines. |
|  | 6b | Control group will receive the same treatment as in the intervention group. However, subject is given press placebos (made by Seirin Corporation) which are identical to the press needles in all aspects except the needle element is lacking. |
| Objectives | 7 | The study aims to establish the benefits of acupuncture as adjunctive therapy in the management of type 2 diabetes mellitus. |
| Trial design | 8 | This is a randomized, double-blinded (patients and practitioner), placebo and two-arm parallel controlled clinical trial. The study will adhere to the CONSORT (Schulz, Altman & Moher, 2010) and STRICTA (MacPherson et a.l, 2010) guidelines (Table 1). |
| Study setting | 9 | The study is planned to be conducted at a public university teaching hospital in Malaysia. |
| Eligibility criteria | 10 | Patients must provide written informed consent before enrolling into the trials.  **Inclusion criteria**   1. Malaysian, age between 30 years and 69 years old with BMI ≤ 40.0 kg/m² 2. Type 2 diabetes mellitus patients with fasting venous plasma glucose (FPG) ≥ 7.0 mmol/L (126 mg/dL) or glycated hemoglobin (HbA1c) of ≥ 6.5% 3. Individuals have had type 2 diabetes mellitus for more than one year 4. Diabetes mellitus under oral anti-diabetic agents on a stable dose over the previous 3 months   **Exclusion criteria**   1. a) Diabetes mellitus under insulin therapy 2. Individuals with other acute or chronic health problems (eg. chronic kidney disease [stage 4 and 5]/heart/liver failure, cancer, cardiovascular disease, stroke, physical disability, mental illness, nephrotic syndrome, decompensated congestive heart failure and oedema on the abdomen) 3. Needle phobia or allergy to adhesive plaster 4. Planning to move out from Malaysia within 4 months’ 5. Being pregnant, planning for pregnancy or lactating women |
| Interventions | 11a | Eligible patients will be randomized in equal proportions between acupuncture group and control group. Both groups will continue with their diabetes regular regimens. The treatment group will receive real acupuncture treatments using press needles (PYONEX ø0.20×1.5mm made by Seirin Corporation) on abdomen area (10 sessions of treatment). The control group will receive press placebos (made by Seirin Corporation) which is identical to the press needle in all aspects except the needle element is lacking. During the intervention, patient’s medications will not be changed. |
|  | 11b | In both groups, participants will be treated twice weekly in the first four weeks then once a week for the next two weeks. In certain circumstances; once-a-week treatment or a three-time per week treatment is permitted. |
|  | 11c | Face-to-face adherence reminder will take place at every treatment sessions. Subsequently, adherence to the protocol is assessed at every follow-up session and evaluation of intervention adherence will be assessed at post intervention. |
|  | 11d | Patients are required to continue with their existing treatment regimen. All medications that are allowed in this study are oral anti-diabetic drugs, hypertensive and hyperlipidemia medications. There will be no change in other treatment as long as patients are clinically stable. |
| Outcomes | 12 | **Primary Outcome Measures**  Difference of homeostasis model assessment-insulin resistance (HOMA-IR) between/within group(s) at week 7 after treatment.  **Secondary Outcome Measures**  i. Health-related quality of life (HRQoL)   1. difference between the two treatment arms at week 3-4 after the treatment 2. difference between the two treatment arms at week 7 after the treatment   ii. Adverse event – difference in the incidence between the two treatment arms at 7 weeks after the treatment |
| Participant timeline | 13 | The primary outcome will be measured at baseline, and after the treatment. The secondary outcome of HRQoL will be measured at baseline, during and after the treatment is completed. Figure 3 shows the schedule for data collection during the 7 weeks of study period*. Data will be collected at the time of recruitment (-0 week), after completion of 5 sessions (week 3/4) and 10 sessions (week 7) of the treatment.  *7 weeks of study period – 6 weeks of treatment and 1 week of follow-up for adverse event after treatment ends. |
| Sample size | 14 | Sixty patients, with 30 participants in each group are required in each group to detect a significant mean difference of 1.23 % HOMA-IR, 1.25% standard deviation [18], 95% confidence level and with a power of 95%. |
| Recruitment | 15 | Subject recruitment is expected to be conducted at a public university teaching hospital in Malaysia from May 2021 until July 2023 by an endocrinologist and a traditional Chinese medicine practitioner through patients’ routine medical check-up schedules. Patients will be provided study brochure and information sheet if they express interest to participate in the study. Upon agreement to participate in the study, patients will be requested to fill up a screening form to assess the eligibility. Type 2 diabetes mellitus will be defined as one abnormal FPG (≥ 7.0 mmol/L (126 mg/dL), random venous plasma glucose (≥ 11.0 mmol/L (200 mg/dL), two hour plasma glucose measurement (≥ 11.0 mmol/L (200 mg/dL) or HbA1c (≥ 6.5%) for screening purpose. It is based on subjects’ self-reported or through their diabetes mellitus medical record, medications packages, and clinic follows up cards. Potential subjects will be invited to attend a blood test screening session. Type 2 diabetes mellitus is confirmed with subject’s HbA1c ≥ 6.5% or morning FPG ≥ 7.0 mmol/L (126 mg/dL) after fasting at least 10 hours according to American Diabetes Association diagnostic criteria. Patients are required to sign a written informed consent form once they fulfill all the study criteria and agree to participate in the study. |
| **Methods: Assignment of interventions (for controlled trials)** | | |
| Allocation: | | |
| Sequence  generation | 16a | Blocked randomization with block size of 2 will be performed in order to minimize between-group differences based on the level of HOMA-IR and BMI. |
| Allocation  concealment  mechanism | 16b | Both the press needle and press placebo are indistinguishable in appearance and they are repacked in identical plastic containers. Every subject is assigned a consecutive number. Sealed opaque envelopes will be used to conceal treatment allocation until the end of the study. |
| Implementation | 16c | If patient express interest to participate in the study, they will be provided study brochure and information sheet. Patients are requested to fill up a screening form to assess the eligibility. Potential subjects are invited to attend a blood screening session. Patients are required to sign a written informed consent form once they fulfill all the study criteria and agree to participate in the study. Patients will be allocated randomly in a 1:1 ratio to treatment or control groups using block randomization. All the press needles and placebos will be repacked in identical plastic containers. They will be consecutively numbered for each subject according to the randomization schedule. The plastic containers will be distributed to the principle investigator at the sites and practitioner(s) will receive the corresponding containers. |
| Blinding  (masking) | 17a | Randomization sequence and allocation will be concealed to all subjects, acupuncturist, laboratory personnel and researchers. |
|  | 17b | To maintain the overall quality and legitimacy of the clinical trial, code breaks should occur only if there is any serious adverse event occurred. The site investigators (Cheok Yean Chin, Ng Ooi Chuan) must record all code breaks with reason on the corresponding CRF. |
| **Methods: Data collection, management, and analysis** | | |
| Data collection methods | 18a | During data collection, all subjects’ anonymity will be maintained. The confidentiality of records and documents that could identify subjects will be protected. The fasting insulin and glucose concentrations will be used to derive the primary outcome of HOMA-IR [fasting serum insulin (μU/ml)×fasting plasma glucose (mmol l-1)/22.5]. The secondary outcomes are changes in HRQoL. Subject’s HRQoL will be assessed using a validated World Health Organization Quality of Life Assessment: Brief Version (WHOQoL-BREF [English/Malay]) questionnaire. The English or Malay versions of this instrument have been validated in Malaysia. For anthropometry measurements, subjects will be measured for weight in kilogram to the nearest 0.1 kilogram, using a digital scale TANITA model HD-382 weighing machine in a standing position; height will be measured using SECA body meter model 208 to the nearest 0.05 cm; BMI is calculated weight in kilogram divided by height in meters squared (BMI=Weight(kg)/Height^2^(m²) and WC will be measured midway between the lowest rib and the iliac crest using SECA measuring tape to the nearest 0.05 centimetre. Socio-demographic information of patients will also be recorded at recruitment. Every participant will be assigned with a coded identification number. The primary outcome of HOMA-IR  will be measured at the time of recruitment (-week 0), and after completion of 10 sessions (week 7) of the treatment. Additionally, secondary outcome of HRQoL will be measured at the time of recruitment (-week 0), after completion of 5 sessions (week 3/4), and 10 sessions (week 7) of the treatment. Any adverse event will be recorded at every visit. |
|  | 18b | In the event that subjects do not turn up for treatment or assessment, they are invited to join the other subsequent session. Once a subject is enrolled or randomized, the researcher will make every reasonable effort to follow the subject for the entire study period. However, the researcher may due to concerns for subject’s safety, stop the study at any time. If the study is stopped early for any reason, subjects will be informed and may be asked to attend for a final visit. The investigators are allowed to use any information obtained up until the time subject withdraw consent. Withdrawal subject will be followed up until the end of the study and every effort must be made to report the observation. |
| Data management | 19 | All study data will be entered into computer by a study investigator (Cheok Yean Chin). All study-related physical files will be stored in a secure place. The data will be kept at Setia Chinese Medical Centre for five years after the data collection. Decoding procedure shall be undertaken if there is any serious adverse event occurred. |
| Statistical methods | 20a | Comparisons of baseline continuous variables between acupuncture and control group will be examined using independent *t* test, while for categorical variables the Chi-squared test will be used. The primary outcome is the change in HOMA-IR from baseline to 7 weeks. The secondary outcome measure is the change of HRQoL from baseline to week 3/4 and week 7. This will be done using a linear mixed model. The model included treatment group (acupuncture or control), time (baseline, week 3/4, and week 7), age, gender, and their interaction as fixed effects. Random intercepts for each subject is also being included in the model to account for individual variability within the data. Intention-to-treat analysis (ITT) will be performed, and all patients enrolled will be included in the analysis, irrespective of compliance. The significant level is set at P<0.05. |
|  | 20b | Not applicable |
|  | 20c | Missing data will be imputed using “last observation carried forward” method. |
| **Methods: Monitoring** | | |
| Data monitoring | 21a | Data monitoring is not needed as this is a postgraduate project (Cheok Yean Chin). Upon completion of data entry / analyses / interpretation, the data will be reported to meet thesis requirement. |
|  | 21b | The trial will not be stopped unless there is any serious adverse event that cause by the trial, if the risks are found to outweigh the potential benefits or when there is conclusive proof of positive and beneficial results. In this case, the investigators will discuss the results of interim-analysis with the Ethics Committee members for Research Involving Human Subjects of Universiti Putra Malaysia. |
| Harms | 22 | All adverse events occurring after entry into the study events will be recorded. Any serious adverse event (SAEs) occur are reported to the principle investigator and the Ethics Committee immediately using SAEs Form. Details about the SAEs that are suspected to cause the adverse event will be evaluated according to National Cancer Institute- Common Terminology Criteria for Adverse Events guidelines based on event intensity (grade 1 - 5). Higher grades mean worst adverse event. |
| Auditing | 23 | The Ethics Committee for Research Involving Human Subjects of Universiti Putra Malaysia will conduct random trial audit, if necessary. |
| **Ethics and dissemination** | | |
| Research ethics approval | 24 | This protocol and the informed consent form have been approved by the Ethics Committee for Research Involving Human Subject of Universiti Putra Malaysia (JKEUPM-2018-294). |
| Protocol amendments | 25 | Not relevant |
| Consent or assent | 26a | Patients are required to sign a written informed consent form before joining the trial. Patients will be given adequate time to read the consent form before signing it. |
|  | 26b | Not relevant |
| Confidentiality | 27 | All study-related information and subject information that contain names or other personal identifiers will be stored at the study site with limited access. In order to maintain subject confidentiality, all laboratory specimens, reports, data collection and patient consent forms will be identified by a coded identification number only. |
| Declaration of interests | 28 | The authors have declared that no competing of interests exists. |
| Access to data | 29 | All investigators will have full access to the complete and final dataset. |
| Ancillary and post-trial care | 30 | Patients enrolled in the study are covered by insurance for any non-negligent harm arises. They will be follow-up for at least 3 months after the intervention. |
| Dissemination policy | 31a | Data will be disseminated through publication in PhD thesis / journals and presentations at national / international seminars / conferences. Patient individual data will be shared with patient, only upon request by the patient. |
|  | 31b | No |
|  | 31c | No |
| **Appendices** | | |
| Informed consent materials | 32 | Sample informed consent form |
| Biological specimens | 33 | Not relevant |
